# Electrical spectroscopy of defect states and their hybridization in monolayer MoS_2_

**DOI:** 10.1038/s41467-022-35651-1

**Published:** 2023-01-03

**Authors:** Yanfei Zhao, Mukesh Tripathi, Kristiāns Čerņevičs, Ahmet Avsar, Hyun Goo Ji, Juan Francisco Gonzalez Marin, Cheol-Yeon Cheon, Zhenyu Wang, Oleg V. Yazyev, Andras Kis

**Affiliations:** 1 Institute of Electrical and Microengineering, École Polytechnique Fédérale de Lausanne (EPFL), CH-1015 Lausanne, Switzerland; 2 Institute of Materials Science and Engineering, École Polytechnique Fédérale de Lausanne (EPFL), CH-1015 Lausanne, Switzerland; 3 Institute of Physics, École Polytechnique Fédérale de Lausanne (EPFL), Lausanne, Switzerland; 4 Department of Materials Science and Engineering, National University of Singapore, Singapore, 117575 Singapore

**Keywords:** Two-dimensional materials, Electronic devices

## Abstract

Defects in solids are unavoidable and can create complex electronic states that can significantly influence the electrical and optical properties of semiconductors. With the rapid progress in the integration of 2D semiconductors in practical devices, it is imperative to understand and characterize the influence of defects in this class of materials. Here, we examine the electrical response of defect filling and emission using deep level transient spectroscopy (DLTS) and reveal defect states and their hybridization in a monolayer MOCVD-grown material deposited on CMOS-compatible substrates. Supported by aberration-corrected STEM imaging and theoretical calculations, we find that neighboring sulfur vacancy pairs introduce additional shallow trap states via hybridization of individual vacancy levels. Even though such vacancy pairs only represent ~10% of the total defect concentration, they can have a substantial influence on the off currents and switching slopes of field-effect transistors based on 2D semiconductors. Our technique, which can quantify the energy states of different defects and their interactions, allows rapid and nondestructive electrical characterization of defect states important for the defect engineering of 2D semiconductors.

## Abstract (web summary)

Deep level transient spectroscopy (DLTS) is an established characterization technique used to study electrically active defects in 3D semiconductors. Here, the authors show that DLTS can also be applied to monolayer semiconductors, enabling in-situ characterization of the energy states of different defects and their interactions.

## Introduction

Two-dimensional (2D) semiconducting materials such as MoS_2_ are considered as one of the most promising material platforms for future electronic devices^1,2^. Observation of high on/off current ratios and reduced standby currents at nm-scale gate lengths, together with high mobility make them promising candidates for extending Moore’s law^3,4^. At the same time, their unique physical properties make them appealing for next-generation applications, including spintronics, valleytronics, twistronics and straintronics, which are considered as frontier replacements for beyond-the-Moore’s era^5–10^. Even though large-scale synthesis of these materials in a scalable fashion has been achieved by metal-organic chemical vapor deposition (MOCVD)^11–14^, the material quality and device performance are lagging behind expectations due to the ubiquitous structural defects^15,16^, which can create unexpected doping^17^, increase charge carrier scattering and introduce hopping transport^18^ as well as degrade the efficiency of optoelectronic devices through nonradiative recombination^19^. To accelerate the process of realizing optimal devices with desirable electrical performance, defects in these materials need to be well understood as a first step towards reducing their concentration and impact on device performance.

There are several techniques available for characterizing defects in 2D materials, with their own advantages and disadvantages. For instance, high-resolution transmission electron microscopy (TEM) and scanning tunneling microscopy (STM) provide direct visualization of atomic defects^15,16,20^. However, the small and localized field of view together with the required material transfer process onto non-conventional (holey or conductive) substrates result in additional complications when compared with a regular device in which the 2D material has undergone significant processing, which could alter its composition and defect concentration. In addition, the inevitable electron beam-induced damage makes TEM a destructive method. Alternatively, optical spectroscopy methods such as Raman^21,22^, X-ray photoelectron^23^, and photoluminescence spectroscopies^24^, as well as electrical characterization techniques like capacitance-voltage (C-V) measurements^25^ can give insights into the presence of disorder in the material integrated into a device, but are often qualitative in nature. The development of a reliable and practical method, capable of performing precise analysis on regular device architectures is required for 2D materials.

Deep level transient spectroscopy (DLTS)^26^, a characterization technique for studying electrically active defects, based on the filling and emptying of trap states via voltage pulses, has been extensively employed for defect characterization of traditional semiconductors due to its extremely high sensitivity and wide detection ranges in the band gap^26–28^. It provides information that can be used for defect identification and fingerprinting, including the defect types (n/p), trap concentrations, activation energies, capture cross sections, emission rates, etc. Despite numerous advantages, this method has so far not been applied to 2D semiconductors. The most common geometry for DLTS measurements is a vertical Schottky diode or a p-n junction in which voltage pulses are used to modulate the width of the depletion region. This is however not possible to achieve in monolayer 2D semiconductors because of their atomic-scale thickness, and as a result, only bulk MoS_2_ has been studied using DLTS^29,30^. The application of this technique to technologically relevant monolayer crystals grown using MOCVD or a similar technique remains unexplored.

Here, we extend the range of applications for DLTS to 2D semiconductors and report DLTS measurements on a single-layer TMDC-based device by employing the metal-insulator-semiconductor (MIS) configuration and adapting the method to the 2D geometry. We find electrical signatures related to sulfur vacancies and detect hybridized defect states due to neighboring sulfur vacancy pairs. These states lie closer to the conduction band edge than the previously considered sulfur vacancy states and have a significant contribution to the density of states and could negatively impact the mobility, on/off ratio and switching slope of field-effect transistors based on 2D semiconductors. Our findings demonstrate that DLTS is a powerful and versatile technique for the non-destructive electrical defect characterization of 2D semiconductors, which could be used for benchmarking the material quality, making it essential for defect engineering and mitigation.

## Results

### DLTS method for monolayer semiconductors

**Fig. 1:**
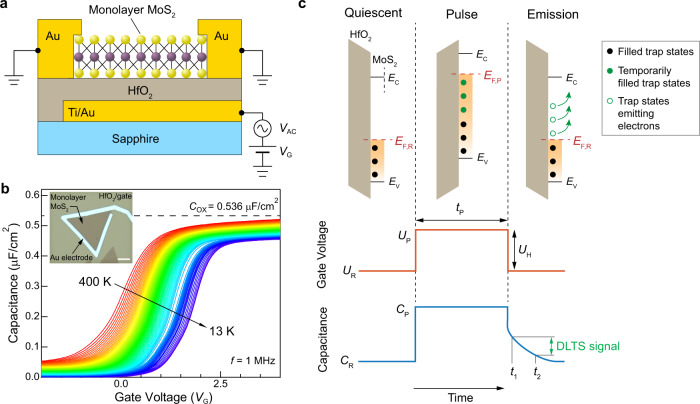
Device configuration for deep-level transient spectroscopy (DLTS) measurements. **a** The metal-insulator-semiconductor (MIS) capacitor device configuration used for capacitance-voltage (C-V) and DLTS measurements. **b** Temperature-dependent C-V curve measured using a 1 MHz, 50 mV oscillating signal *V*_AC_ superposed on the DC gate voltage *V*_G_. The horizontal, dotted line marks the saturating capacitance from the oxide (*C*_OX_). The inset shows the optical image of the MIS capacitor device. The scale bar is 10 µm long. **c** A schematic diagram showing the band alignment, the carrier interaction with trap states, and the voltage and capacitance change during the DLTS measurement. The orange-shaded regions represent the MoS_2_ band below the Fermi level *E*_F_. The green arrows represent the carrier emission process. In panel **c,** *E*_C_ and *E*_V_ represent respectively the energy level of conduction band and valence band, while *E*_F,R_ and *E*_F,P_ are Fermi levels in the reference (quiescent) and pulsed states, respectively. The pulse parameters are respectively the reference voltage *U*_R_, the pulse voltage *U*_P_, the pulse height *U*_H_, and the pulse width *t*_P_, and *C*_R_ and *C*_P_ represent the device capacitances in the reference (quiescent) and pulsed states. The DLTS signal is measured as the capacitance difference between the rate window *t*_1_ and *t*_2_.

Our single-crystal MoS_2_ is grown on a c-plane sapphire substrate in a hot-wall MOCVD reactor. Supplementary Fig. 1a shows its triangular shape with a large lateral size and sharp crystal edges. Raman spectroscopy confirms the single-layer thickness of the as-grown material (Supplementary Fig. 1b). Figure 1a shows the metal-insulator-semiconductor (MIS) structure used throughout this work. Our device is fabricated on top of the sapphire substrate to eliminate any background-related charges and noise and to minimize stray and background capacitance. We use a gate stack composed of a local Ti/Au (2 nm/30 nm) bottom gate and a high-κ dielectric HfO_2_ (30 nm thick) to effectively modulate the electrical field and tune the conductivity of the MoS_2_ flake. Finally, we pattern the top contact composed of 80 nm of Au contacting the perimeter of the MoS_2_ crystal (Fig. 1b inset) to achieve ohmic-like contacts for effective charge carrier injection.

Prior to DLTS measurements, we first perform a basic capacitance-voltage (C-V) characterization to evaluate the device performance in the steady state. We sweep the back-gate voltage (*V*_G_) in the ±5 V range with a 50 mV, 1 MHz oscillating signal (*V*_AC_) connected in series, while measuring the capacitance between the bottom gate and the top contact (Fig. 1a, b). Our MOCVD-grown MoS_2_ device exhibits n-type conductivity with a threshold voltage (*V*_TH_) of 0.65 V at room temperature, taken as the *V*_G_ with half of the oxide capacitance (1/2 *C*_OX_) as previously suggested^31,32^. The rapid capacitance saturation indicates an effective Fermi level (*E*_F_) tuning by the gate voltage. The small frequency dispersion and the low series resistance (Supplementary Note 2) indicate high-quality devices.

Traditional DLTS measurements rely on the modulation of the depletion region in a semiconductor device (Supplementary Note 3 and refs. 26, 30), typically a vertical Schottky diode. The atomic-scale thickness of monolayer 2D semiconductors however prevents the formation of the depletion width in the vertical direction. Instead, by using the MIS configuration shown in Fig. 1a, we can modulate the trap filling and extend the DLTS method to monolayer semiconductors by tuning the surface Fermi level by the back-gate voltage *V*_G_^33,34^. Another disadvantage of traditional DLTS geometries based on a vertical Schottky diode is their inability to probe devices in the accumulation regime, since a forward-biased device would carry current. This is not the case for MIS devices where tuning the Fermi level will result in trap filling in both the depletion and accumulation regions.

**Fig. 2:**
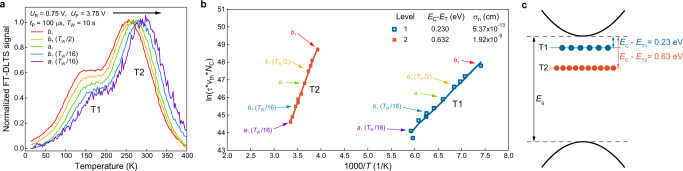
Defect levels in single-crystal MoS_2_ characterized by DLTS. **a** Normalized Fourier transform DLTS signal measured using different correlation functions, showing two types of defect states (T1 and T2). Here, ${b}_{1},\;{b}_{1}({T}_{{{{{{\rm{W}}}}}}}/2),\;{a}_{1},\;{b}_{1}({T}_{{{{{{\rm{W}}}}}}}/16),\;{a}_{1}({T}_{{{{{{\rm{W}}}}}}}/16)$ are 5 correlation functions used as examples to demonstrate peak shifts with different rate windows. The reference voltage *U*_R_ is fixed at 0.75 V, pulse voltage *U*_P_ at 3.75 V, pulse width *t*_P_ at 100 μs, and the period width *T*_W_ at 10 s. **b** Arrhenius fits obtained from panel a, showing the two detected trap states. Here, the table in the inset lists the activation energy *E*_C_-*E*_T_ and the capture cross section *σ*_n_ of T1 and T2 trap states, obtained from the slope and intersect of the two Arrhenius curves. **c** A schematic diagram showing the relative energy positions (*E*_T1_ and *E*_T2_) and trap concentrations (indicated by the number of circles) of the two detected defect states in the band gap. *E*_g_ is the band gap energy of monolayer MoS_2_.

The modification of the DLTS method for studying monolayer semiconductors is illustrated in Fig. 1c. The device is first held in a quiescent depletion condition, with constant reference bias (*U*_R_) and capacitance (*C*_R_) values. By applying a voltage pulse (*U*_P_) with a height *U*_H_ and duration *t*_P_, the Fermi level (*E*_F,R_) is raised to a shallower position (*E*_F,P_), allowing the temporary filling of trap states between *E*_F,R_ and *E*_F,P_, resulting in an increased capacitance, up to *C*_P_. The reference voltage (*U*_R_) and the pulse voltage (*U*_P_) determine, respectively the lower and upper boundary of the energy range within which we can detect the trap states. At the end of the pulse, the filled states start emitting electrons with a time constant (*τ*) and an emission rate (*e*_n_), as indicated by the green arrows, resulting in an exponential capacitance decay. The difference $\triangle C=C\left({t}_{1}\right)-C({t}_{2})$ between capacitances measured at different times *t*_1_ and *t*_2_ is the DLTS signal^26^, with a rate window defined by *t*_1_ and *t*_2_. Changing the temperature modifies the trap emission rates, giving rise to temperature dependence in the DLTS spectra such as the one shown in Fig. 2a and Supplementary Fig. 5, with characteristic peaks related to individual trap levels. Here, we use the Fourier transform (FT) DLTS technique in which the time-dependent capacitance decay is expressed in terms of Fourier coefficients^35^.

In order to quantitatively relate the defect properties to the temperature-dependent DLTS signal, we first derive (Supplementary Note 5) a modified Arrhenius function for the 2D geometry (Eq. (1) and Fig. 1c) to describe how the temperature (*T*), the trap activation energy (*E*_C_−*E*_T_), and the capture cross section (*σ*_n_) influence the carrier emission rate and lifetime:

1 $${{{{{\rm{ln}}}}}}\left(\tau \cdot {T}^{\frac{3}{2}}\cdot {K}_{2{{{{{\rm{D}}}}}}}\right)=\frac{{E}_{{{{{{\rm{C}}}}}}}-{E}_{{{{{{\rm{T}}}}}}}}{{k}_{{{{{{\rm{B}}}}}}}}\cdot \frac{1}{T}-{{{{{\rm{ln}}}}}}\left({\sigma }_{{{{{{\rm{n}}}}}}}\right)$$

Here, *E*_C_ is the conduction band minimum, *E*_T_ is the energy level of the trap state, *k*_B_ is the Boltzmann constant, and *K*_2D_ is a constant value independent of temperature and trap energy, equal to 2.74 × 10^16^ cm^−1^·s^−1^·K^−3/2^. By plotting ${{{{{\rm{ln}}}}}}(\tau \cdot {T}^{\frac{3}{2}}\cdot {K}_{2{{{{{\rm{D}}}}}}})$ as a function of 1/*T*, we can directly obtain *E*_C_-*E*_T_ and *σ*_n_ values through a linear fit. Equation (1) is similar to the 3D Arrhenius function (Supplementary Note 5), but with a different temperature exponent, due to the dimensional difference in thermal velocity (${v}_{{{{{{\rm{th}}}}}}}$) and the effective electron density of states (*N*_C_). The capture cross-section *σ*_n_, representing the capability of a trap state to capture electrons and holes, has a dimension of length for a single-layer device, rather than area as for a bulk semiconductor. In general, *σ*_n_ is a material parameter which can be influenced by multiple factors such as the defect size and Coulomb charging^36^.

### Trap state-level determination by DLTS

To identify the optimal pulsing conditions and investigate different emission conditions, we first carry out a series of DLTS measurements at room temperature with various pulse parameters (Supplementary Note 6). Subsequently, we perform the DLTS measurement as a function of temperature to extract the trap characteristics using Eq. (1). Figure 2a and Supplementary Fig. 7 show the normalized temperature scan of a series of transient signals at different rate windows, accessed by 11 correlation functions (${a}_{1},\;{b}_{1},\;{b}_{1}\left({T}_{{{{{{\rm{W}}}}}}}/2\right),$ etc.) which correspond to the following Fourier coefficients obtained from the Fourier series of the capacitance transient^35^:

2 $${a}_{{{{{{\rm{n}}}}}}}\left(\tau \right)=\frac{2\Delta C}{{T}_{{{{{{\rm{W}}}}}}}}{e}^{-\frac{{t}_{0}}{\tau }}\left(1-{e}^{-\frac{{T}_{{{{{{\rm{W}}}}}}}}{\tau }}\right)\frac{1/\tau }{\frac{1}{{\tau }^{2}}+{n}^{2}{\omega }^{2}}$$

and

3 $${b}_{{{{{{\rm{n}}}}}}}(\tau )=\frac{2\Delta C}{{T}_{{{{{{\rm{W}}}}}}}}{e}^{-\frac{{t}_{0}}{\tau }}(1-{e}^{-\frac{{T}_{{{{{{\rm{W}}}}}}}}{\tau }})\frac{n\omega }{\frac{1}{{\tau }^{2}}+{n}^{2}{\omega }^{2}}$$

where *n* is the order of the Fourier series, *T*_W_ the measurement window, *ω* = 2π/*T*_W_ and ∆*C* is the amplitude of the capacitance transient.

We can observe two prominent FT-DLTS peaks in Fig. 2a, related to two distinct trap states in our MoS_2_ device that we label T1 and T2. Taking the *τ* value for each correlation function and the corresponding peak temperature, we plot the Arrhenius curves, and extract the trap activation energy and capture cross section for both states as shown in Fig. 2b. We find that T1 corresponds to a shallower state, located 0.230 eV below *E*_C_ while T2 is related to a deeper state at 0.632 eV below *E*_C_, Fig. 2c. The relative trap concentrations are related to peak heights^26^, indicating the prevalence of T2 over T1. Due to its higher prevalence and similarity with previously reported trap energy levels^15,16^, we tentatively assign the T2 trap state to sulfur vacancies (V_S_). The extracted capture cross-section of T2 is 1.92 × 10^−9^ cm, similar in size to a vacancy site and comparable to the square root of the *σ*_n_ value obtained from its exfoliated, bulk counterpart^30^. On the other hand, the energy level of the T1 state does not match with those commonly associated with single (V_S_) or double (V_S2_) sulfur vacancies^15,16^ and therefore requires further investigation outlined here using STEM imaging and density-functional theory (DFT) calculations.

### Trap state energy linewidth determination by DLTS

**Fig. 3:**
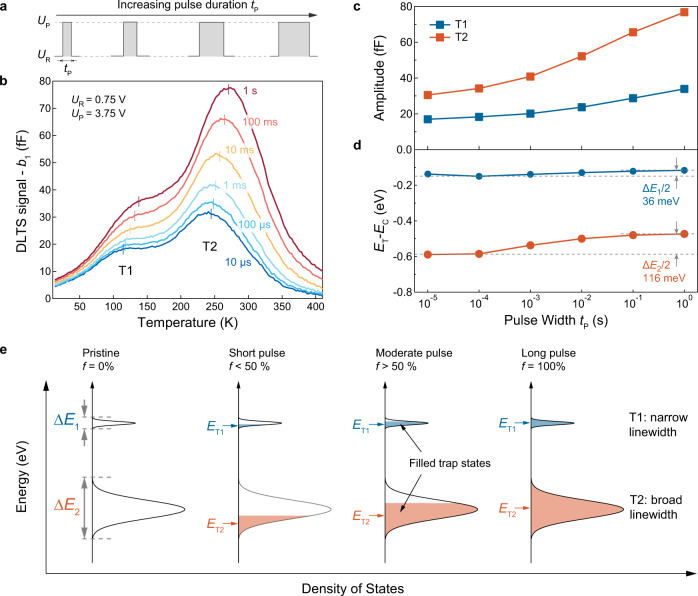
Defect level linewidth and control of defect filling. **a** A simple scheme and **b** the DLTS temperature sweep at various pulse width *t*_P_ values. Here, the reference and pulse voltages are fixed at 0.75 V and 3.75 V respectively. **c** Change of peak amplitudes and **d** measured trap energies with respect to *t*_P_. Each data point is obtained from the two peak positions in panel b. The gray dash line in panel d marks the shifting of trap energies from short to long pulses. **e** A schematic diagram showing the measured *E*_T_ values changing with the trap-filling ratio (*f*) for T1 and T2 states. The blue and orange shaded regions represent the part of T1 and T2 density of states (DOS) filled by charge carriers. ∆*E*_1_ and ∆*E*_2_ represent the total energy linewidth for T1 and T2 defects respectively.

In addition to quantifying the trap level position inside the band gap, DLTS can also provide additional defect fingerprinting information in the form of the trap state energy linewidth. It can be extracted from the DLTS measurements by modifying the voltage pulse width *t*_P_ and/or the voltage pulse amplitude *U*_P_, resulting in different levels of trap filling reflected in the DLTS signal amplitude, shown on Fig. 3 and Supplementary Figures 8 and 9. Amplitudes of both T1 and T2 peaks (Fig. 3b) increase with *t*_P_ (Fig. 3c) as a result of increased trap state filling. By performing Arrhenius fitting for each of the DLTS curves in Fig. 3b, we can extract the variation of trap activation energy *E*_C_-*E*_T_ with *t*_P_ (Fig. 3d). The effective trap energy level remains practically constant for the T1 state (36 meV shift) but exhibits a larger shift closer to the band edge for the T2 peak (116 meV shift). Similar trends are also observed with increased *U*_P_ values but the influence is less significant (Supplementary Fig. 9).

We explain this behavior by considering the trap state energy linewidth (∆*E*) as illustrated on Fig. 3e. The trap activation energy *E*_C_−*E*_T_ extracted from DLTS measurements corresponds to the average energy of the filled trap states shown as the shaded regions. For completely filled traps, this would correspond to the ideal *E*_C_−*E*_T_ value, while partially filled trap states would result in *E*_C_−*E*_T_ values shifted to lower energies^37^. The energy linewidth ∆*E* of a given trap state can then be estimated as the double of the *E*_C_−*E*_T_ shift from the values obtained for empty (*f* = 0, short *t*_P_) to complete trap-filling conditions (*f* = 100%, long *t*_P_), as shown in Fig. 3e. We expect that states with a narrower linewidth would show a smaller variation with *t*_P_, such as in the case of T1. States with a wider distribution would be more sensitive to *t*_P_. This is the case for the T2 trap which shows a saturating energy shift for the longest pulse duration (*t*_P_ = 1 *s*) achievable in our setup. We extract from our measurements effective linewidths of ∆*E*_1_ = 72 ${{{{{\rm{meV}}}}}}$ for the T1 state and ∆*E*_2_ = 232 meV for the T2 state. Such linewidth estimations further reinforce DLTS as an important defect characterization technique since this information cannot be accessed *via* STEM or electrical transport measurements.

### Defect characterization by STEM

**Fig. 4:**
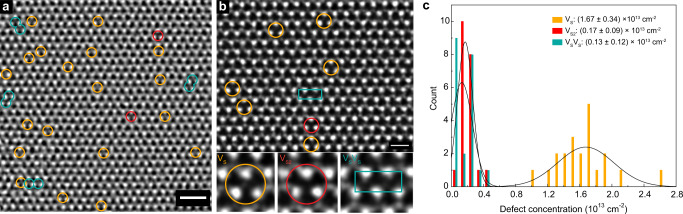
Atomic defects characterized by scanning transmission electron microscope (STEM). **a**, **b** Filtered STEM image showing different types of defects. Orange and red circles highlight single (V_S_) and double (V_S2_) sulfur vacancy defects, respectively, while the turquoise green box marks two V_S_ right next to each other (V_S_V_S_). **c** Histogram (bar shape) and corresponding Gauss fit (solid line) of V_S_, V_S2_, V_S_V_S_ concentrations calculated from 21 pristine regions of the as-grown single crystal. The scale bars in panels a and b are respectively 1 nm and 0.5 nm long.

To investigate the origin of the two defect states detected by DLTS measurements, we perform aberration-corrected STEM on the as-grown MoS_2_. The wide-field annular dark field (ADF)-STEM image in Fig. 4a shows the atomic structure of free-standing monolayer MoS_2_, where Mo (*Z* = 42) atoms appear brighter in comparison to the S2 (*Z* = 32) atoms owing to their Z-contrast dependency. To minimize possible errors caused by the localized field of view, we perform ADF-STEM imaging on 21 pristine regions. In all regions, we observe only sulfur vacancy defects in our material and do not observe any Mo vacancies or antisite defects that were previously reported for CVD-grown materials^15^, implying excellent growth conditions in our MOCVD method. In Fig. 4a, b, we label the three most common vacancy configurations: isolated single sulfur vacancy (V_S_), isolated double sulfur vacancy (V_S2_), and two single sulfur vacancies next to each other (V_S_V_S_), as highlighted by orange, red, and turquoise green colored circles and boxes, respectively. The concentration histogram and the average defect densities of V_S_, V_S2_, and V_S_V_S_ measured from 21 pristine regions are presented in Fig. 4c. These values are of the same order of magnitude as in exfoliated samples, indicating the high quality of our MoS_2_ crystal^38^. In addition, we observe an apparent sulfur vacancy creation under the electron-beam irradiation, allowing us to estimate the intrinsic defect concentrations (Supplementary Note 9)^39^. These STEM results confirm that the two defects inferred from the DLTS measurements are both related to sulfur vacancies.

### DFT modeling and hybridized defect states

**Fig. 5:**
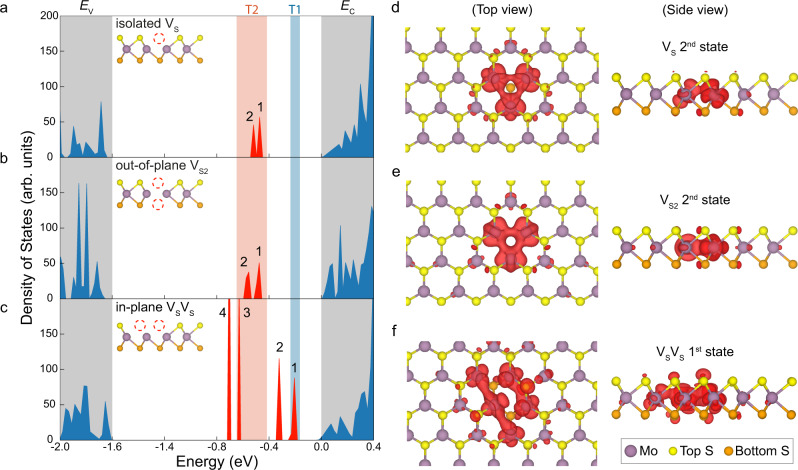
Defect energy levels and hybridization calculated by density functional theory (DFT). **a–c** Calculated density of states (DOS) of **a**, an isolated single sulfur vacancy (V_S_), **b**, double sulfur vacancies (V_S2_), and **c**, a neighboring sulfur vacancy pair on the same lattice plane (V_S_V_S_). Inset shows the side view of the vacancy positions in MoS_2_ atomic lattice. The shaded gray regions mark out the valence and conduction bands. The calculated in-gap defect states are indicated in red, and the blue and orange shaded regions are respectively the T1 and T2 defect states measured by DLTS. Here, the band gap energy *E*_g_ is predicted to be 1.68 eV using the Perdew-Burke-Ernzerhof (PBE) functional. The two states for V_S_ or V_S2_ belong to a single level but include the spin-orbit coupling. **d**–**e** Top and side view of the charge density distribution of the 2nd in-gap state for **d**, V_S_ and **e**, V_S2_ defects as marked in panels **a**–**b**. **f** Top and side view of the charge density distribution of the 1^st^ in-gap states for the neighboring V_S_V_S_ pair shown in panel **c**.

Our DFT calculations show that single sulfur vacancies (V_S_) (Fig. 5a), the dominant defect type identified by ADF-STEM imaging, exhibit energy levels 0.5–0.6 eV below E_C_, in line with the T2 energy measured by DLTS and other reported calculations^15,16^. We note that the band gap energy is systematically underestimated by the Perdew-Burke-Ernzerhof (PBE) functional (1.68 eV) and therefore, the deviation of ~0.1 eV between DLTS and DFT is within the error range (for more discussion please refer to Supplementary Note 10). Strikingly, with top and bottom sulfur atoms missing, the double sulfur vacancy defect (V_S2_) has nearly the same energy levels as V_S_ inside the gap. In addition to the in-gap state, V_S2_ is also predicted to have another defect level located inside the conduction band (Supplementary Fig. 12)^15^, which, however, is outside the detection range of our DLTS technique^26^. Despite the large hybridization-induced splitting of around 0.6 eV that pushes one of the states into the conduction band, we still observe similar defect energy positions and overlapping density of states (DOS) with V_S_ (Fig. 5a, b) inside the gap. We note that the charge densities associated with both V_S_ and V_S2_ defect states (Fig. 5d, e) show a comparable localized behavior with the three closest Mo atoms displaying the largest density. Small differences can be observed in the side view: while the charge density of the V_S_ defect states shows a slight localization preference towards the side of the missing atom, the V_S2_ density is mirror-symmetric along the Mo atoms. Furthermore, we do not observe meaningful differences in the Mo-S bond length (2.41 Å) modulation near the defects as in both cases the Mo-S bonds lengths are within the range of 2.38–2.45 Å. This makes it difficult to distinguish between these two trap types, as also revealed by the apparent peak width and the relatively large full-width at half maximum (FWHM) in DLTS measurement (Fig. 2a). These results clearly verify the origin of the T2 state to be V_S_ and V_S2_ defects, as well as excluding their possible relation with the T1 state.

According to the DFT calculations, four in-gap hybridized defect states are created for the first nearest neighbor sulfur vacancy pairs (V_S_V_S_, 1NN), with their energies distributed between 0.2 eV and 0.7 eV below *E*_C_ (Fig. 5c). The splitting between the hybridized defect states is approximately 0.4 eV, making it smaller than the splitting observed in V_S2_ and hence leading to additional in-gap states close to the conduction band edge. Interestingly, the charge density of, for example, the first (Fig. 5f) and third (Supplementary Fig. 15d) in-gap states clearly show a different localization pattern in comparison to the V_S_ and V_S2_ defects. Most importantly, we can see that a large part of the charge density is now also delocalized over the neighboring sulfur atoms and even more distant Mo atoms. Although the local bond length modulations show minor differences with the V_S_ and V_S2_ defects, we note that the minimum Mo-S bond length (2.36 Å) is observed exactly in the V_S_V_S_ configuration. Evidently, the reduced interaction of the two in-plane as opposed to the two out-of-plane vacancies lead to a type of hybridization, where 4 distinguishable in-gap states appear. In addition, we also note that the energies of these hybridized states highly depend on the distance between the neighboring sulfur vacancies (Supplementary Fig. 14). Continuously increasing the distance would lead to the convergence of these four defect levels into two levels resembling the DOS of V_S_. Comparing to the measured *E*_C_-*E*_T_ value of 0.230 eV by DLTS, we attribute the T1 trap to the shallower doublet state created by V_S_V_S_ that has been directly observed in STEM (Fig. 4a, b). We expect a slight contribution of V_S_V_S_ to the T2 trap as well, but their influence is less significant compared to the more abundant isolated V_S_. We exclude other defect types based on the discussion in Supplementary Note 10.

### Sulfur vacancy creation by Ar plasma probed by DLTS

In order to verify the origin of DLTS signal from sulfur vacancies, we performed additional measurements on a similar MoS_2_ MIS device that is exposed to mild Ar plasma to create a higher concentration of defects (Supplementary Note 11). After 4 seconds of mild plasma treatment, we observe an increased DLTS signal of around 25%, as well as the right shift of the DLTS peaks towards higher temperature, which indicates an increase of sulfur vacancy concentration, especially the creation of neighboring sulfur vacancies (Supplementary Fig. 16). These results demonstrate a correlation of V_S_ concentration variation with the DLTS signals and further confirm the attribution of the DLTS signal to sulfur vacancies.

Moreover, additional experiments using transferred metal contacts demonstrate the same dominant DLTS peak associated with the T2 (V_S_ and V_S2_) state, which excludes the contact fabrication as the source of defects in our monolayer MoS_2_ MIS devices (Supplementary Note 12). Besides, a control experiment using the metal-insulator-metal (MIM) structure further eliminate the influence of oxide or parasitic capacitances on our DLTS spectra (Supplementary Note 13).

## Discussion

Defects in 2D materials are usually considered independent of each other and their influence on electrical performance are studied individually. In monolayer MoS_2_, the most common defect, the single sulfur vacancy (V_S_) is predicted to create only one deep state (*E*_C_−*E*_T2_ ~ 0.5 eV) in the band gap, as also confirmed experimentally by STM spectroscopy^38^. In addition to this state, we also resolve a previously overlooked shallower defect level (*E*_C_−*E*_T1_ ~ 0.2 eV) created by the hybridization between neighboring pairs of V_S_ (V_S_V_S_). Since these states lie closer to the conduction band edge, they will significantly shift the threshold voltage and decrease the subthreshold slope in field-effect transistors. In addition, compared to the single defect level created by V_S_, the four hybridized defect levels associated with V_S_V_S_ pairs can substantially increase the chance of defect-assisted recombination, which could lead to diminished quantum yield in optoelectronic devices. Even though their concentration corresponds to only 10% of isolated V_S_ (Fig. 4c), the neighboring V_S_V_S_ pairs give rise to a distinct DLTS peak (T1) with 60% of the T2 peak height (Fig. 2a), indicating a significantly larger density of in-gap states than for their isolated counterparts. This increased DOS is due to the hybridization of neighboring sulfur defects as seen from the charge density plots (Fig. 5f and Supplementary Figure 14-15). Therefore, to achieve high quality devices with minimum density of states and narrowest energy linewidth, defect hybridization needs to be largely avoided. For that, a minimum distance of ~ 8.44 Å (4NN in Supplementary Fig. 14) needs to be maintained between sulfur vacancies, corresponding to a maximum defect concentration of 3.5 × 10^13^ cm^−2^.

To summarize, we demonstrate the adaptation of the DLTS measurement technique to a single-layer, MOCVD-grown MoS_2_ crystal using a CMOS compatible device geometry. This allows us to identify a shallow state (T1) induced by the hybridization of neighboring sulfur vacancy pairs on the same lattice plane, supported by STEM imaging and DFT calculations. Capability of energy linewidth detection by DLTS measurements is also demonstrated, confirming the narrow bandwidth of the hybridized shallow state, as well as considerable energy broadening of V_S_ traps. Considering the high sensitivity of DLTS measurements and its applicability to capacitor-based devices, such as field-effect transistors, our study provides a pathway for a comprehensive understanding of atomic defects and their electrical properties in 2D semiconductors in the single-layer limit. We expect the future application of DLTS technique in other 2D materials to enrich the defect library, to reveal unexplored defect characteristics and to enable effective defect engineering and mitigation. With the rapid progress of 2D materials towards future electronic device applications, the DLTS technique can serve as an efficient tool for defect characterization and device improvement.

## Methods

### Material synthesis

The MoS_2_ single crystals are grown on c-plane sapphire chips in a 2-inch hot-wall tube reactor using the metal-organic chemical vapor deposition method. We first anneal the sapphire chips at 1000 °C in air for 2 h to create atomically smooth terraces for epitaxial growth^40^. Prior to growth, 0.26 mol/L sodium chloride (NaCl) solution is spin-coated to suppress the nucleation and promote the crystal growth^13^. Molybdenum hexacarbonyl (Mo(CO)_6_) and hydrogen sulfide (H_2_S) are used as the gas precursors to allow maximal control. Using 70 sccm Ar as the carrier gas, they are mixed with a molar ratio of 1:2009 in the tube furnace. The reaction takes place at 850 °C and is terminated with a cut-off of Mo(CO)_6_ precursor after 30 min of growth time. The furnace is then cooled down naturally to 150 °C with a continuous H_2_S supply. The pressure is maintained at 850 mbar by a pressure regulator throughout the whole growth process. The optical microscopy image and Raman spectroscopy (Supplementary Fig. 1) confirm the monolayer thickness of the as-grown MoS_2_.

### Sample transfer and STEM imaging

Atomic-scale annular dark-field scanning transmission electron microscopy (ADF-STEM) imaging of monolayer MoS_2_ is performed using an aberration-corrected STEM (FEI Titan Themis 60–300), which is equipped with a Schottky X-FEG bright source, Cs double corrector and a monochromator. Before the STEM imaging, the as-grown MOCVD material is transferred onto a commercial silicon nitride TEM grid in DI water using the PMMA/gel-pak assisted method. Afterward, the grid is immersed in hot acetone and annealed in high vacuum for 10 h at 250 °C to remove the polymer resists. Imaging is performed at a low acceleration voltage of 80 kV, which is below the displacement knock-on damage of MoS_2_. The probe current is set to 18 pA for all the experiments. The semi-convergence angle of the electron probe is 20 mrad and at a 185 mm camera length, the angular range of the higher angular annular dark field (HAADF) detector is 49.5–198 mrad. Both images and series are acquired using short dwell time (8 µs) for 512 × 512- pixels. Each image is captured within an exposure time of < 3 s and an e-beam dose rate of < 10^6^ e^−1^nm^−2^s^−1^ to minimize the beam damage. Images from series (Supplementary Figs. 10 and 11) are aligned manually for the drift correction and used to calculate the S defect concentrations. To remove the electron probe-tails, images are processed using double Gaussian filtering.

### MIS capacitor device fabrication

Sapphire wafer, instead of SiO_2_/Si, is chosen as the device substrate due to its excellent insulating property, minimizing stray capacitance. The bottom gate is patterned on the wafer-scale using photolithography, followed by the deposition of 2 nm Ti and 30 nm Au using an e-beam evaporator. The insulating layer is composed of 30 nm of hafnium oxide (HfO_2_) grown via atomic layer deposition (ALD) using TEMAH (tetramethylammonium hydroxide) and water as precursors. After that, we use the PMMA/gel-pak assisted method to transfer the MOCVD-grown MoS_2_ flakes onto the patterned substrate. Polymer residues are then dissolved in acetone and further removed by high vacuum annealing at 250 °C for 8 h. Finally, the top contact is patterned by e-beam lithography, and 80 nm Au as contact is deposited by e-beam evaporation. The contact between metal and MoS_2_ flake is improved by annealing the devices at 200 °C for 3 h before the electrical measurements.

### C-V characterization

C-V characterization is performed in high vacuum after in situ annealing at 120 °C for more than 30 h to remove any surface absorption or contamination. The frequency-dependent C-V curves (Supplementary Fig. 3) are measured at room temperature using a Keysight E4980A precision LCR meter. The oscillation voltage is fixed at 50 mV with varied frequencies from 1 kHz to 1 MHz. The back gate voltage is swept forward and backward in the range of ± 5V with a sweeping rate of 20 mV/s.

The temperature-dependent C-V curves (Fig. 1b and Supplementary Fig. 2) are measured using a Boonton 7200 capacitance meter embedded in the PhysTech DLTS system. We perform a C-V measurement every 5 K as the temperature is increased from 12 K to 400 K. The oscillation frequency is fixed at 1 MHz to improve the signal-to-noise ratio, and the sweeping range and speed are the same as described above.

### DLTS characterization

The DLTS measurements are performed on a PhysTech FT-1030 system. Capacitance transient signals are digitalized through numerical Fourier transformation, which allows the acquisition of 28 discrete Fourier coefficients within a single temperature scan. Comparing to the conventional boxcar method, FT-DLTS guarantees a higher accuracy through noise suppression and automatic control of measurement parameters^35^. Here, due to the monolayer nature of the material, the transient signal is on the scale of tens or hundreds of femtofarad (fF), resulting in a relatively low signal to noise ratio comparing to bulk semiconductors. Each temperature sweep of the DLTS is performed from 400 K to 13 K with an interval of 2.5 K. Variation of each pulsing parameter (Fig. 3 and Supplementary Fig. 9) is obtained during one temperature sweep to guarantee identical measurement conditions.

### DFT calculations

Our first-principles calculations are performed at the density-functional theory level with spin-orbit coupling included as implemented in VASP^41^. The exchange-correlation potential is approximated by the generalized gradient approximation (GGA) using the semilocal Perdew-Burke-Ernzerhof (PBE) functional^42^. Electron-core interactions are described through the projector augmented wave (PAW) method^43,44^, while Kohn-Sham wave functions are expanded in a plane-wave basis set with a cutoff on the kinetic energy of 400 eV. The integration over the Brillouin zone is carried out using a 3 × 3 × 1 k-point mesh. All structures are subjected to periodic boundary conditions using a 5 × 5 supercell geometry, while a vacuum layer of 10 Å perpendicular to the monolayer is used to prevent interaction between replica images. Atomic positions and lattice constants are optimized using the conjugate gradient method, where the total energy and atomic forces are minimized. The convergence criterion for energy is chosen to be 10^−5^ eV and the maximum force acting on each atom is less than 0.01 eV/Å relaxation. For all the plotted figures, we set zero energy at the conductance band minimum to easily identify and compare the defect level positions.

### Ar plasma treatment

The Ar plasma treatment is performed using a PICO low-pressure plasma system from Diener electronic. Before treating the target device, a series of plasma tests are conducted on the as-grown MoS_2_ sample by varying the plasma power (15–30 W), the Ar gas flow rate (5–30 sccm), and the treatment duration (1–30 s). Raman spectroscopy is used to assess the material quality after the plasma treatment (similar to ref. 45), which allows us to obtain a mild and non-destructive plasma condition for the device treatment.

The mild plasma is generated by dispersing a 21 W RF power in a 6-inch-diameter vacuum chamber with a 15 sccm of Ar flow, where the MoS_2_ device is kept for 7 seconds (3 second plasma rising time + 4 second exposure time). The MoS_2_ MIS device used here for plasma irradiation is from another chip fabricated together with the devices presented in Figs. 1–3. The C-V and DLTS measurement are performed immediately after the Ar plasma irradiation and an in situ annealing at 120 °C for more than 30 h.

### Transfer of metal contacts

80 nm Au contacts with a designed pattern are deposited on top of clean SiO_2_/Si substrate using an e-beam evaporator, and then picked up by a small piece of poly-dimethylsiloxane (PDMS) capped with 15% weight of poly-propylene carbonate (PPC) in chloroform. Afterwards, the stack is transferred on top of patterned MoS_2_/HfO_2_/Au/sapphire substrate using a transfer stage capable of precise alignment. The detailed transfer process and optical microscope images are shown in Supplementary Fig. 17.

## Supplementary information

Supplementary Information

## Data Availability

The data that support the findings of this study are available in Zenodo at 10.5281/zenodo.7389794.
