# Supplementary material for: Electrical spectroscopy of defect states and their hybridization in monolayer MoS2
Source: Nat Commun. 2023 Jan 3;14:44. doi: 10.1038/s41467-022-35651-1 (PMC9810731; doi:10.1038/s41467-022-35651-1)
Supplement: Supplementary file 1 — Supplementary Information [file 41467_2022_35651_MOESM1_ESM.pdf]

Supplementary Information

for

Electrical spectroscopy of defect states and their hybridization in monolayer MoS_2

Yanfei Zhao[1,2], Mukesh Tripathi[1,2], Kristiāns Čerņevičs[3], Ahmet Avsar[1,2,4], Hyun Goo Ji[1,2], Juan Francisco Gonzalez Marin[1,2], Cheo-Yeon Cheon[1,2], Zhenyu Wang[1,2], Oleg V. Yazyev[3], Andras Kis[1,2*]

[1]*Institute of Electrical and microengineering, École Polytechnique Fédérale de Lausanne (EPFL), CH-1015 Lausanne, Switzerland*
[2]*Institute of Materials Science and Engineering, École Polytechnique Fédérale de Lausanne (EPFL), CH-1015 Lausanne, Switzerland*
[3]*Institute of Physics, École Polytechnique Fédérale de Lausanne (EPFL), Lausanne, Switzerland*
[4]*Department of Materials Science and Engineering, National University of Singapore, Singapore 117575, Singapore*

**Correspondence should be addressed to: Andras Kis, andras.kis@epfl.ch*

Supplementary Note 1: Basic material characterization

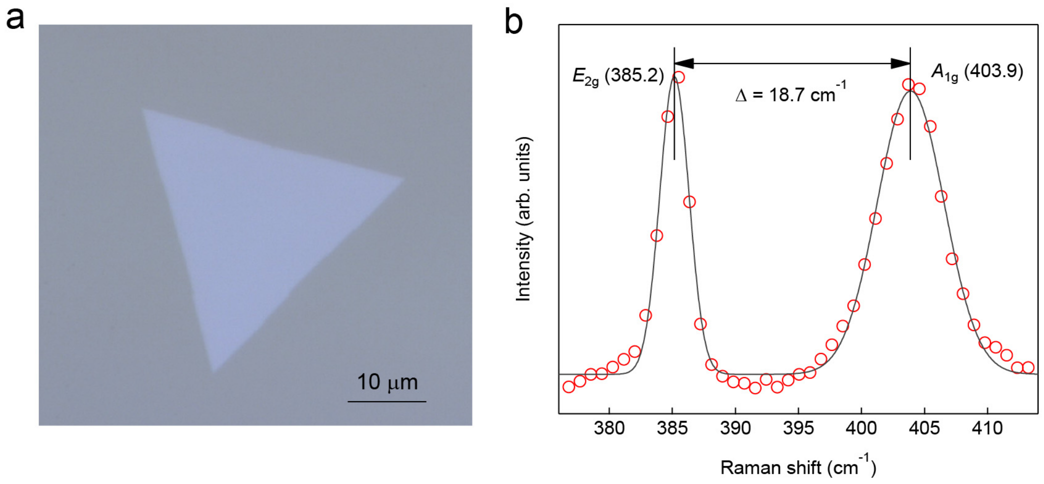

Supplementary Figure 1. Basic material characterization. a) Optical microscope image and b) Raman spectroscopy of an as-grown single-crystal MoS_2 on sapphire using a 532 nm laser.

Supplementary Note 2: C-V characteristics of the single-layer MoS_2 device

We measured a dielectric constant (ε_r) of 18.17 for our ALD HfO_2 from several reference metal-insulator-metal (MIM) devices with different contact areas. This high ε_r value reveals an excellent quality of the dielectric.

In the C-V measurement of Fig. 1b, we calculate the threshold voltage (V_{TH}) of 0.65 V at room temperature, taken as the V_G with half of the oxide capacitance (1/2 C_{OX}). At this position, the MoS_2 quantum capacitance (C_Q) is equal to the oxide capacitance (C_{OX})[1,2]. Using the forward (V_{TH_f}) and backward (V_{TH_b}) threshold voltages, we obtained the room temperature hysteresis to be 0.35 V (Supplementary Figure 2a). In addition, the forward and backward hysteresis is around 0.3-0.6 V at varied temperatures (Supplementary Figure 2b-c), indicating a small amount of interface disorder. At different oscillation frequencies (Supplementary Figure 3a), C-V curves saturate rapidly with almost identical slopes without a significant capacitance drop. This indicates a homogenous potential distribution over the whole flake due to the surrounding configuration of the metal contact[3,4]. Moreover, our device exhibits an excellent metal-semiconductor contact with a series resistance of a few kΩ (Supplementary Figure 3b), taken as the accumulation resistance in the C-V curve[5].

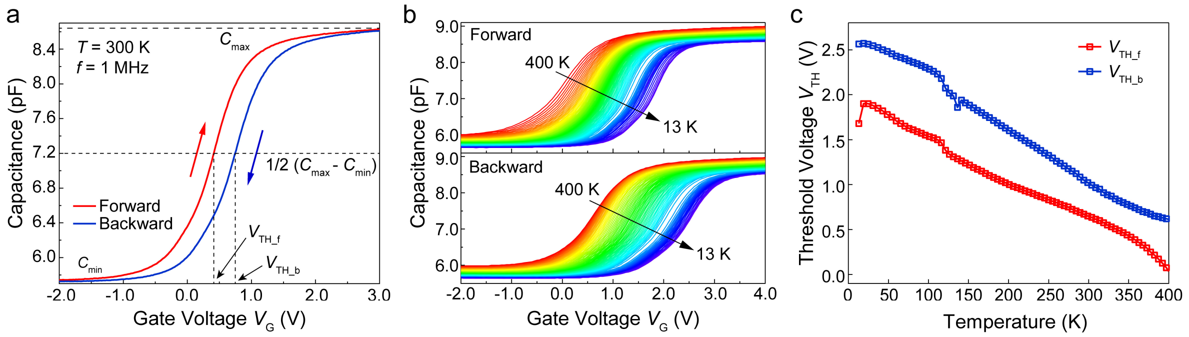

Supplementary Figure 2. C-V measurements. a) Forward and backward sweep of the C-V curve at 300 K. The hysteresis is 0.35 V, measured at the middle point of capacitance. b) Temperature-dependent C-V measurement swept in forward (top panel) and backward (bottom panel) directions. c) The threshold voltage V_{TH} with respect to the measurement temperature. In panels a-b, the gate voltage V_G is swept in the range of ±5 V, and the oscillation voltage V_{AC} is fixed at 50 mV with a 1 MHz frequency. Sweeping speed ΔV_G are respectively 20 mV/s in panel a and 50 mV/s in panel b.

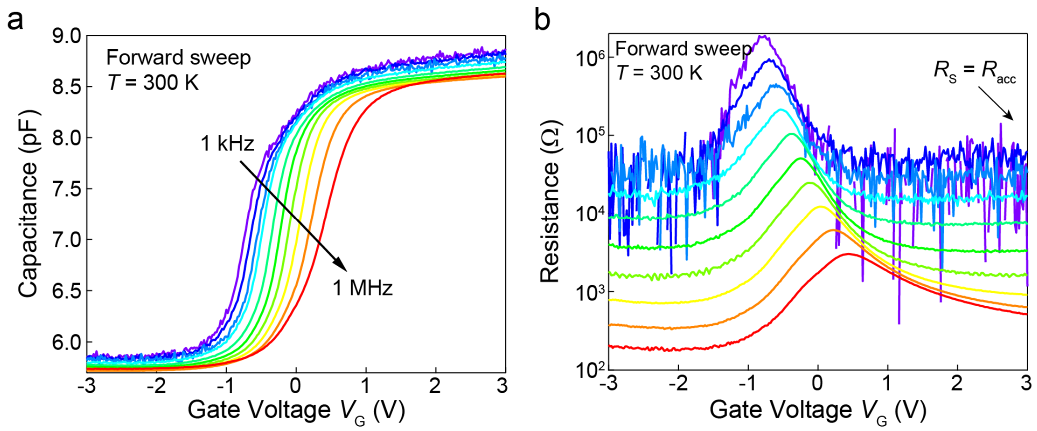

Supplementary Figure 3. Frequency-dependent C-V measurements. a) The capacitance-voltage (C-V) curve and b) resistance-voltage (R-V) curve measured at different oscillation frequencies. We show here the forward sweeping data from -5 V to 5 V with 20 mV step. The series resistance R_S can be taken from the accumulation region of the R-V curve as previously suggested in Supplementary Reference 5, which is pointed out by the arrow in panel b. In all measurements, the oscillation amplitude V_{AC} is fixed at 50 mV.

Supplementary Note 3: DLTS model for a bulk semiconductor device

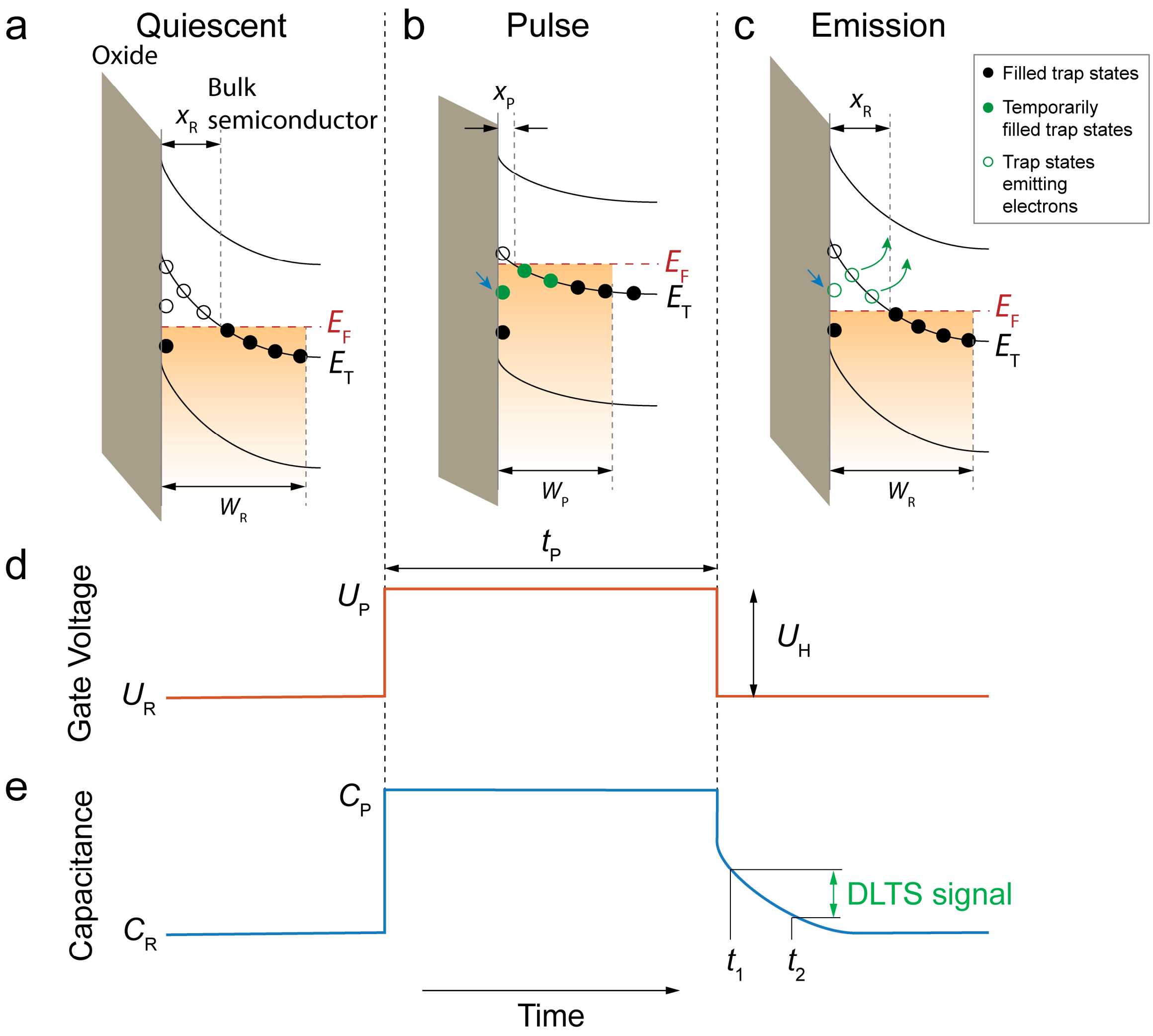

Supplementary Figure 4. Traditional DLTS configuration for an n-type bulk semiconductor. a to c) demonstrate respectively the quiescent state before pulsing, the instant trap filling process during a pulse, and the trap emission process after the pulse. d) Gate voltage and e) capacitance change during the DLTS measurement. In panel a, a constant reference voltage $\boldsymbol{U_R}$ is applied on the back gate voltage and thus creates a depletion region $\boldsymbol{W_R}$ between the interface and the quasi-neutral region. Trap states are filled by electron carriers in the region $\boldsymbol{x_R} < \boldsymbol{x} < \boldsymbol{W_R}$, and are empty in the region $\boldsymbol{0} < \boldsymbol{x} < \boldsymbol{x_R}$, depending on the relative position of trap level $\boldsymbol{E_T}$ and Fermi level $\boldsymbol{E_F}$. During the voltage pulse in panel b, the band bending is relieved and the depletion width ($\boldsymbol{W_P}$) is shortened, which allows an additional region ($\boldsymbol{x_P} < \boldsymbol{x} < \boldsymbol{x_R}$) of trap states to be filled by electrons. In panel c, the gate voltage returns back to $\boldsymbol{U_R}$, leading to the restoration of band bending and depletion width to the initial status. The trap states in the region $\boldsymbol{x_P} < \boldsymbol{x} < \boldsymbol{x_R}$ will start emitting electrons, leading to a capacitance transient recorded by the DLTS instrument. Here, the orange shaded regions represent the MoS_2 band below $\boldsymbol{E_F}$. The black solid, green solid, and green hollow circles respectively represent the trap states, which are filled, temporarily filled, and emitting electrons. The green arrows demonstrate the carrier emission process. As can be observed directly from the band bending, the structure is kept at depletion during the whole process and there is very little carrier accumulation at the interface. Though interface trap states are also filled by electrons as pointed out by the dark blue arrow in panels b-c, the transient signal is dominated by the bulk traps spatially located in the region $\boldsymbol{x_P} < \boldsymbol{x} < \boldsymbol{x_R}$.

Supplementary Note 4: Decay of the transient signal under various temperatures

Here in Supplementary Figure 5, we plot the decay of the transient signal under various temperatures for the data set shown in Fig. 2a. The transient signals are highly dependent on temperature, which can be easily explained by Supplementary Equation 2, where the trap emission rate increases exponentially with temperature. The measured DLTS signal at a certain temperature corresponds to $\Delta C = C(t_1) - C(t_2)$. Therefore, at high temperature (400 K) the transient decays very fast and relaxes before the time window (t_1 and t_2), resulting in almost zero ΔC value. In contrast, the low emission rate at cryogenic temperature (13 K) would lead to a slow transient decay, which expands along the time scale and thus gives a similar small ΔC within t_1 and t_2. In-between, the DLTS signal reaches the local maximum at around 150 K and 250 K, indicating a correspondence between the trap emission rate and the time window defined by the correlation functions (Eq. 2-3).

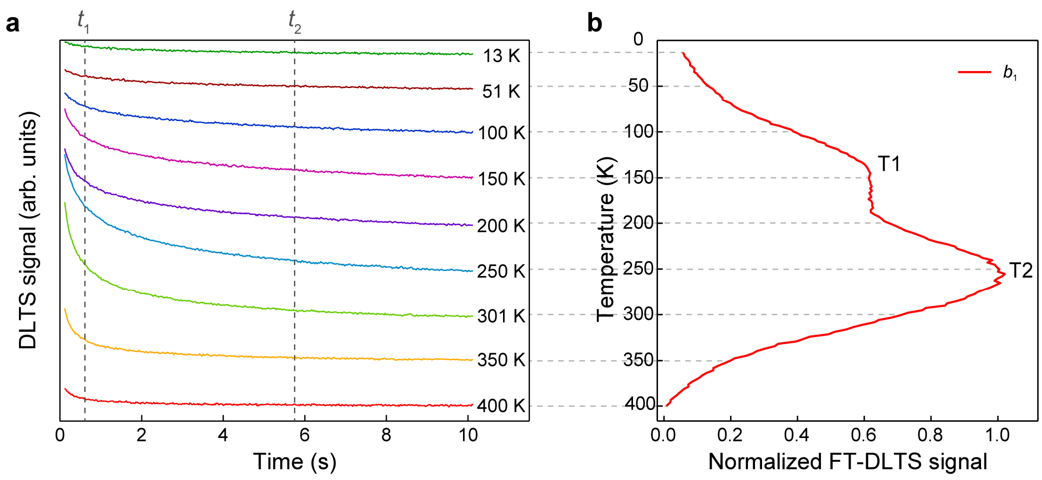

Supplementary Figure 5. DLTS signal changing with temperature. a) The transient decay over time under various temperature conditions. b) The normalized Fourier transform DLTS signal measured using b_1 correlation function or the indicated time window t_1 and t_2 shown in panel a. Two types of defect states are observed with peak transients at around 150 K and 250 K, respectively.

Supplementary Note 5: Arrhenius functions for bulk and single-layer devices

Comparing Fig. 1c and Supplementary Figure 4, we can identify two main differences between the single-layer and bulk devices. First, for single-layer devices, carriers involved in emission

are confined in only one layer, therefore retaining only the energy (E) degree of freedom. In contrast, carrier trapping and emission in bulk semiconductor gives information in both the energy (E) and the width (x) scale, allowing identification of different trap states according to their distance from the interface[6]. Secondly, bulk devices are usually operated in depletion mode ($U_{\mathrm{R}} < U_{\mathrm{P}} < V_{\mathrm{TH}}$), while single-layer devices are tuned from depletion to accumulation ($U_{\mathrm{R}} < V_{\mathrm{TH}} < U_{\mathrm{P}}$). This allows the detection of trap states in a wider energy range in single-layer devices. Except for the above differences from the band diagram, the thermal velocity v_{th} and the effective electron density of states N_{C} are also divergent for bulk and single-layer materials, which can result in slightly different Arrhenius functions as will be described below.

First, the capacitance transient can be described as an exponential decay for a single trap state[6],

$$C(T) - C_{\mathrm{R}} = -\Delta C \cdot e^{-e_{\mathrm{n}} \cdot t} = -\Delta C \cdot e^{-t/\tau} \tag{1}$$

where the carrier emission rate (e_n) and time constant (τ) as a function of the temperature (T) can be expressed by:

$$\frac{1}{\tau} = e_{\mathrm{n}} = \sigma_{\mathrm{n}} \cdot v_{\mathrm{th}} \cdot N_{\mathrm{C}} \cdot e^{-\frac{E_{\mathrm{C}} - E_{\mathrm{T}}}{k_{\mathrm{B}} T}} \tag{2}$$

where σ_{n} is the capture cross section, v_{th} is the thermal velocity, N_{C} is the effective electron density of states, E_{C} is the conduction band minimum, E_{T} is the trap energy level, and k_{B} is the Boltzmann constant. Here, v_{th} and N_{C} for bulk and single-layer materials can be expressed as below:

$$v_{\mathrm{th}}(3D) = \sqrt{\frac{3k_{\mathrm{B}}T}{m_{\mathrm{ce}}^*}} \;;\; v_{\mathrm{th}}(2D) = \sqrt{\frac{2k_{\mathrm{B}}T}{m_{\mathrm{ce}}^*}} \tag{3}$$

$$N_{\mathrm{C}}(3D) = 2\left(\frac{2\pi \cdot m_{\mathrm{de}}^* \cdot k_{\mathrm{B}}T}{\mathrm{h}^2}\right)^{\frac{3}{2}} \;;\; N_{\mathrm{C}}(\mathrm{2D}) = \frac{8\pi \cdot m_{\mathrm{de}}^* \cdot k_{\mathrm{B}}T}{\mathrm{h}^2} \tag{4}$$

Combining Supplementary Equations 2-4, we can obtain the Arrhenius functions for a bulk semiconductor as below:

$$\ln\left(\tau \cdot T^2 \cdot \frac{4k_B{}^2}{h^3}\sqrt{\frac{6\pi^3 m_{de}^*{}^3}{m_{ce}^*}}\right) = \frac{E_C - E_T}{k_B} \cdot \frac{1}{T} - \ln(\sigma_n) \quad \text{(3D Arrhenius)} \tag{5}$$

$$\text{or} \quad \ln(\tau \cdot T^2 \cdot K_{3D}) = \frac{E_C - E_T}{k_B} \cdot \frac{1}{T} - ln(\sigma_n) \quad \text{(3D Arrhenius)} \tag{6}$$

and the modified Arrhenius function for 2D materials as below:

$$\ln\left(\tau \cdot T^{\frac{3}{2}} \cdot \frac{8\sqrt{2}\pi \cdot m_{de}^* \cdot k_B{}^{\frac{3}{2}}}{m_{ce}^*{}^{\frac{1}{2}} \cdot h^2}\right) = \frac{E_C - E_T}{k_B} \cdot \frac{1}{T} - \ln(\sigma_n) \quad (2D\ Arrhenius) \tag{7}$$

$$or \quad \ln\left(\tau \cdot T^{\frac{3}{2}} \cdot K_{2D}\right) = \frac{E_C - E_T}{k_B} \cdot \frac{1}{T} - \ln(\sigma_n) \quad (2D\ Arrhenius) \tag{8}$$

where Supplementary Equations 6 and 8 are simplified with constant K_{3D} and K_{2D} values, which are calculated to be $1.56 \times 10^{21}\ \mathrm{cm^{-2} \cdot s^{-1} \cdot K^{-2}}$ and $2.74 \times 10^{16}\ \mathrm{cm^{-1} \cdot s^{-1} \cdot K^{-\frac{3}{2}}}$ respectively, if taking the effective mass as $m_{de}^* = m_{ce}^* = 0.48\ m_0$[7]. Except for the different K values, the temperature factor of $\ln(T^2)$ for 3D and $\ln(T^{3/2})$ for 2D on the left side could also result in systematic errors if directly using the 3D Arrhenius function on our single-layer device for energy and cross section calculation. In addition, the unit of capture cross section σ_n has changed from cm^2 in 3D structure into cm in 2D, which physically means the possibility of electron carriers being trapped in width (W) scale rather than area scale ($W \times t$).

Supplementary Note 6: DLTS isothermal measurement at room temperature

To find the optimal pulsing parameters and to investigate different emission conditions, we performed a series of isothermal measurements at room temperature by varying the pulse width t_P, reference voltage U_R, and pulse voltage U_P. As shown on Supplementary Figure 6a, the DLTS signal first increases logarithmically with the extended pulse duration, then reaches a plateau when t_P > 6.8 μs, and grows slightly when t_P exceeds 100 ms. These two stages of growth signify respectively the charging of the MoS_2 traps at the interface and the slow-responsive oxide traps[8]. Therefore, we choose t_P = 100 μs as our default parameter to guarantee

a maximum charging of atomic defects at the interface, while avoiding unnecessary oxide charging.

In Supplementary Figure 6b-d, we further explore its dependence on the applied gate voltage. First, we fix the $U_{\mathrm{P}}/E_{\mathrm{F,P}}$ at strong accumulation and vary $U_{\mathrm{R}}/E_{\mathrm{F,R}}$ from a deep to shallow position to enable the detection of trap states in a varied energy range (Supplementary Figure 6b). We observe the appearance of a DLTS peak when U_{R} is 0.6 V ($U_{\mathrm{R\_peak}}$), indicating the dominant trap states located near $E_{\mathrm{F,R\_peak}}$ in the band gap. Similar behavior is also observed in Supplementary Figure 6c when fixing the pulse height U_{H} and varying U_{R} and U_{P} simultaneously. Compared with the temperature-dependent DLTS measurement shown in Fig. 2, we can attribute the isothermal peak to T2 or V_S and V_{S2}. In Fig. 2, we use a U_{R} value giving the maximum DLTS signal in the isothermal measurements (Supplementary Figure 6).

In Supplementary Figure 6d, we investigate the dependence of the DLTS signal on the pulse voltage U_{P}. As sketched in the inset, we fix the $U_{\mathrm{R}}/E_{\mathrm{F,R}}$ near the position of T2 ($U_{\mathrm{R\_peak}}/E_{\mathrm{F,R\_peak}}$) and increase the $U_{\mathrm{P}}/E_{\mathrm{F,P}}$ from a weak depletion to a strong accumulation region. In this way, we not only allow the trap filling from deep to shallower positions, but also increase the filling rate of deep traps. We observe that the DLTS signal arises at U_{P} = 2 V and continue growing with the increase of U_{P}, revealing the partial filling of trap states at low pulse voltage.

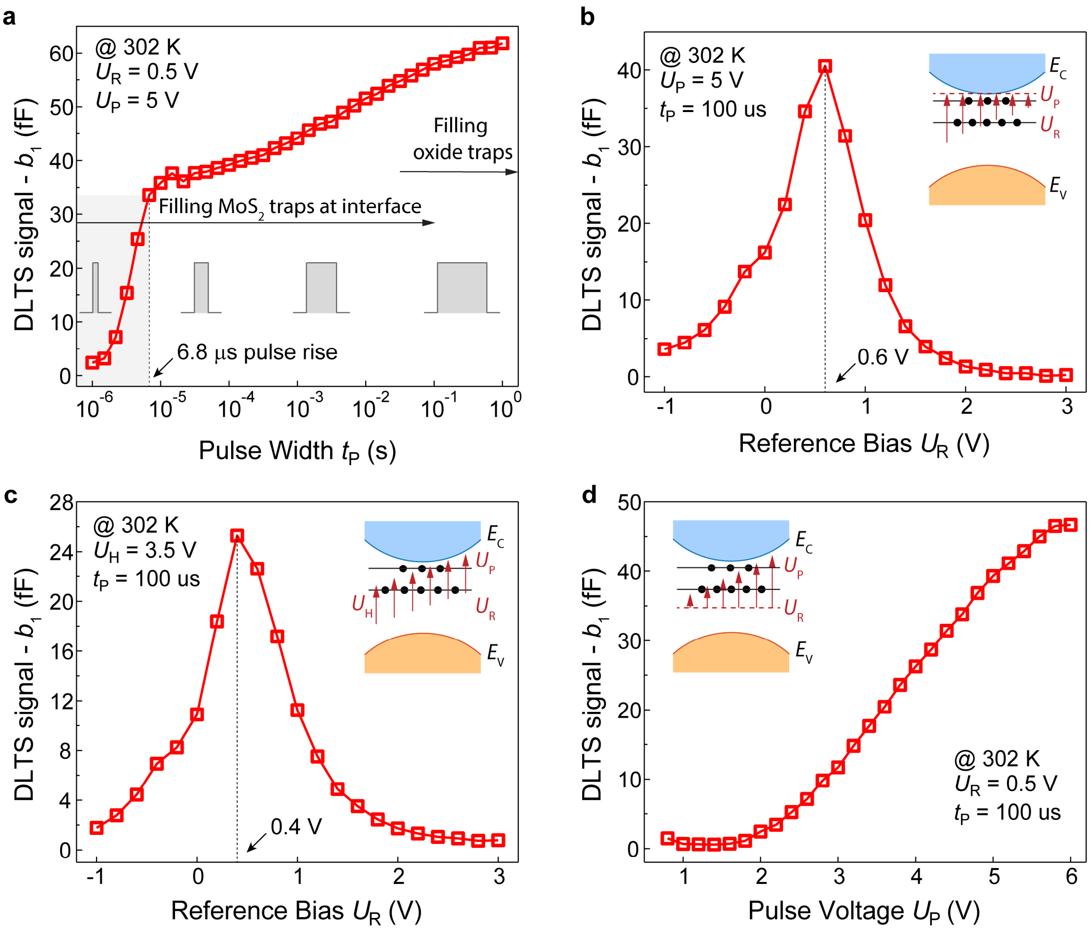

Supplementary Figure 6. DLTS isothermal measurement performed at 300 K. DLTS signal change with a) the pulse width $\boldsymbol{t_P}$ while fixing the voltage conditions, b) the reference voltage $\boldsymbol{U_R}$ while fixing the $\boldsymbol{U_P}$, c) $\boldsymbol{U_R}$ and $\boldsymbol{U_P}$ together while fixing the pulse height $\boldsymbol{U_H}$, and d) the pulse voltage $\boldsymbol{U_P}$ while fixing the $\boldsymbol{U_R}$.

Supplementary Note 7: Normalized DLTS temperature scan

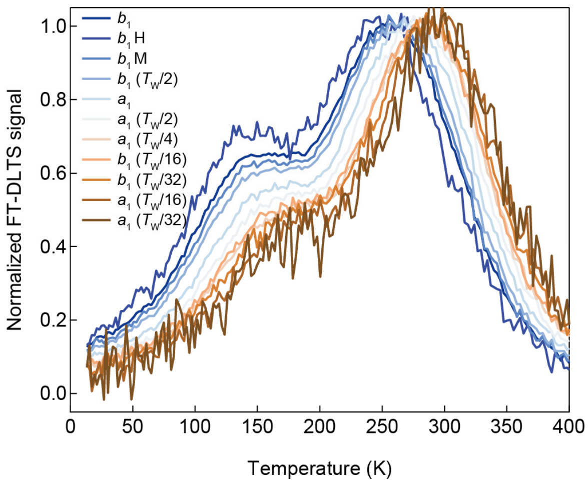

Supplementary Figure 7. Normalized Fourier transform DLTS signal measured using different correlation functions. Here shows the 11 correlation functions with good signal to noise ratios. They are used to plot the Arrhenius curves shown in Fig. 2b and to calculate the $\boldsymbol{E_C - E_T}$ and $\boldsymbol{\sigma_n}$ values.

Supplementary Note 8: Tuning the trap-filling ratio by t_P and U_P

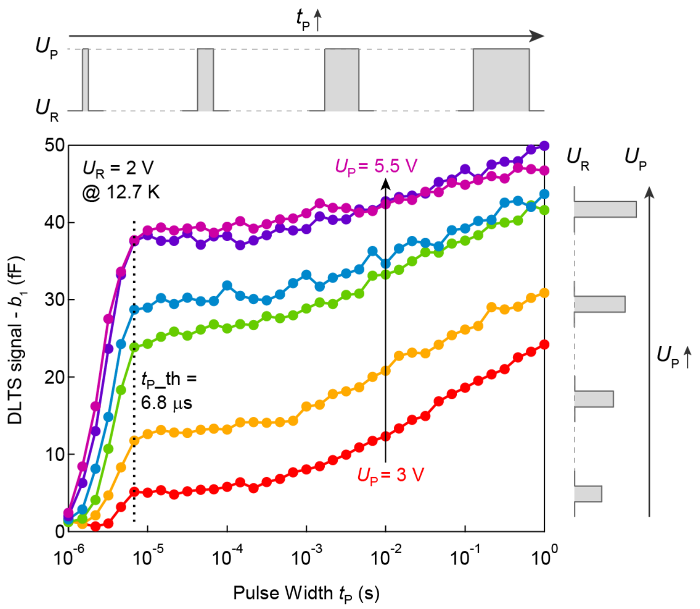

Supplementary Figure 8. DLTS isothermal measurement under various t_P and U_P conditions. $b_1 - t_P$ curves for different U_P values measured at 12 K. The period width T_W is 10 s in all measurements. The DLTS amplitude increases along with t_P and U_P, indicating a rising trap-filling ratio. There is an obvious critical point at t_P_th = 6.8 µs for the pulse to rise up. Below t_P_th, the interface traps are not sufficiently charged while above that, the filling ratio depends more significantly on U_P values. When U_P is higher than 5 V and t_P longer than 6.8 µs, the DLTS amplitude is almost constant and only slightly increases when t_P > 10 ms. However, this isothermal behavior and the filling ratio can also be temperature-dependent, requiring further investigation.

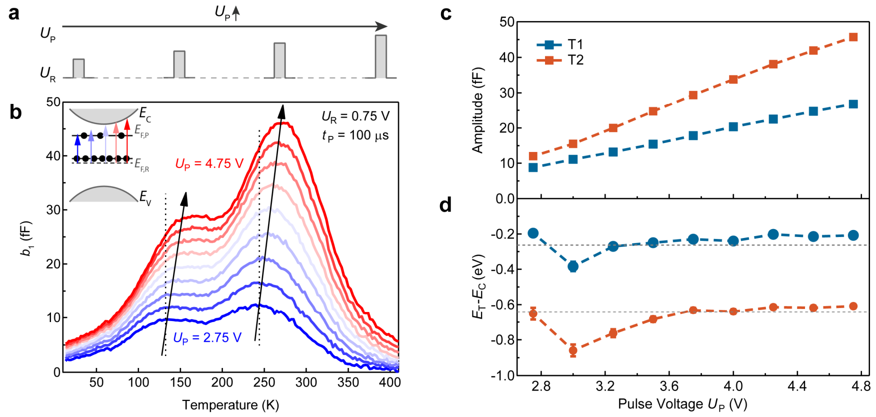

Supplementary Figure 9. DLTS measurement under various U_P conditions. a) A simple scheme and b) the DLTS temperature sweep at various pulse voltage U_P values. For all curves, the reference voltage U_R and the pulse width t_P are fixed at 0.75 V and 100 µs respectively. c) Change of peak amplitudes and d) measured trap energies with respect to U_P. Each data point is obtained from the two peak positions in panel b.

Supplementary Note 9: STEM characterization

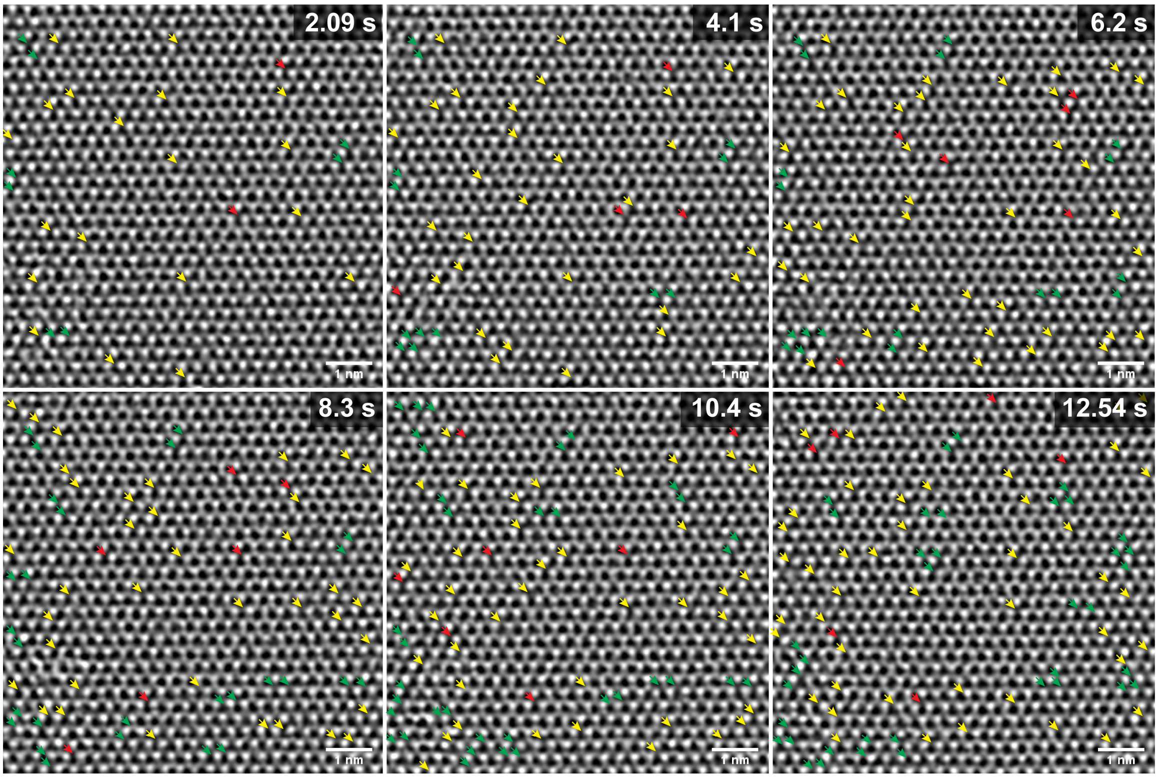

Supplementary Figure 10. Sulphur vacancy (V_S) creation under electron beam irradiation during STEM characterization. To probe the evolution of S vacancies under continuous electron-beam irradiation, a time series was recorded from a pristine region. ADF-STEM images here show the first six frames acquired from the series. The increasing number of SVs are clearly visible in the images. Yellow, red, and green arrows point out V_S, V_{S2}, and V_SV_S defects respectively.

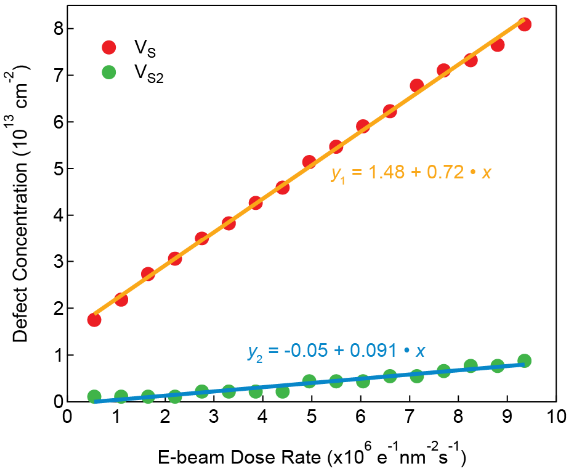

Supplementary Figure 11. Sulphur vacancy concentration with respect to the accumulated e-beam dose rate. Through a linear fit, we are able to eliminate the systematic error from beam damage. The slope and intercept of the linear fitting represent respectively the defect generation rate and the intrinsic defect concentration before beam damage.

Supplementary Note 10: DFT calculations

Although the Perdew-Burke-Ernzerhof (PBE) functional has been shown to systematically underestimate the band gap size[9], it has been widely used in MoS_2 defect calculations[10–12] partly due to the large supercell sizes (5×5, 6×6) needed to investigate isolated defects and hence the too high computational cost to employ hybrid functions that would lead to a more precise band gap. Furthermore, by utilizing a small supercell (3×3) we observed that the defect levels located close to the conduction band edge are only shifted at most by ±0.1 eV with respect to conduction band minimum when comparing the PBE with a hybrid functional (HSE06) calculation hence confirming the validity of our PBE calculations.

Additionally, we have included spin-orbit coupling (SOC) in our calculations as the lack of inversion symmetry in monolayer MoS_2 lifts the spin degeneracy of the electronic bands with valance band displaying large spin splitting of around 150 meV[13]. This inclusion of SOC also leads to the lifting of the empty degenerate in-gap energy level of single (V_S) and double (V_{S2}) sulfur vacancy and hence we observe two close laying levels as opposed to one degenerate level[10]. All of our investigated defect systems are charge neutral.

According to the STEM and DLTS results in the main text, it is obvious that the T2 trap located at 0.632 eV below E_C is created mainly by single sulfur vacancy (V_S) with a slight influence of double sulfur vacancy V_{S2} and V_S pairs. However, for the other subordinate trap (T1) with an $E_C - E_T$ value of 0.230 eV, there is also a possibility of sulfur substitution by H or O, or adatom. They are invisible from the STEM investigation but can create electrically active states in the band gap (Supplementary Figure 13). Here, H adatom, possibly created from water molecules, has a similar activation energy to T1. However, in case of any H adsorption, they would be removed during the in-situ annealing process prior to measurement. On the other hand, the substitutional isoelectronic O atoms form a strong bond to Mo and are considered to be common even after annealing, but in turn provide no in-gap defect states[14]. Besides, antisite

defects such as S_{Mo} and Mo_{S2} are directly excluded here because there is no evidence of their existence in our materials, as confirmed by STEM imaging.

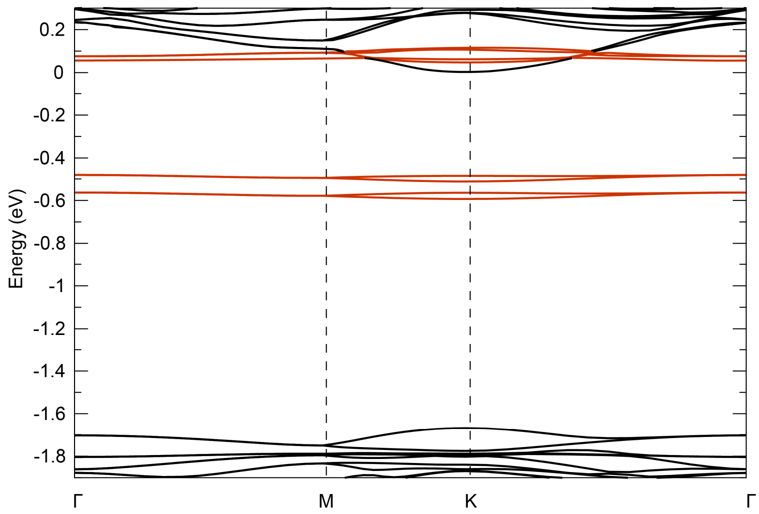

Supplementary Figure 12. Band structure of the double sulfur vacancy V_{S2}. As marked in red color, four defect bands are visible in the diagram, with two states in the conduction band and two mid-gap states exhibiting similar energy levels as V_S.

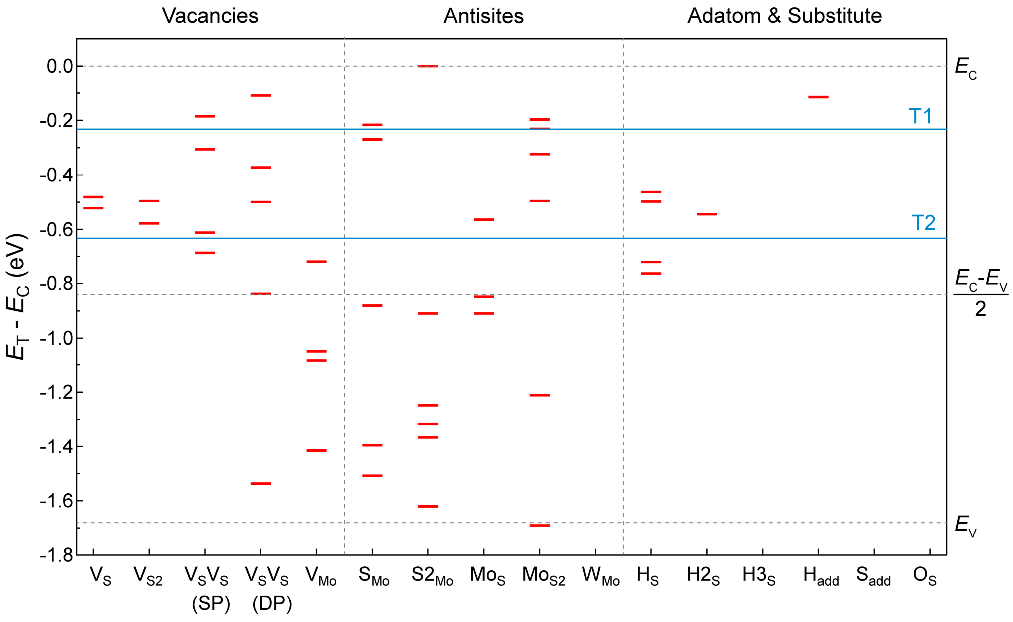

Supplementary Figure 13. Defect energy levels for various defect types in MoS_2 from DFT calculations. The investigated vacancy defects include single sulfur vacancies (V_S), double sulfur vacancies (V_{S2}), first nearest neighboring single sulfur vacancy pairs (V_SV_S), and molybdenum vacancies (V_{Mo}). Here, we calculated two relative positions of V_SV_S: in the same plane (SP) and different planes (DP). Antisite defects include single sulfur replacing molybdenum (S_{Mo}), double sulfur replacing molybdenum ($S2_{Mo}$), molybdenum replacing single sulfur (Mo_S), molybdenum replacing double sulfur (Mo_{S2}), and tungsten replacing molybdenum (W_{Mo}). Light atom adsorption or substitution defects are invisible under STEM but could exist on the MoS_2 surface. H_S, $H2_S$, $H3_S$, and O_S respectively represent one hydrogen atom, two hydrogen atoms, three hydrogen atoms and one oxygen atom filling a single sulfur vacancy. H_{add} and S_{add} respectively represent hydrogen and sulfur adatom on top of a sulfur atom. Note: $H3_S$ values are taken from Supplementary Reference 15 because they are not convergent in our calculation.

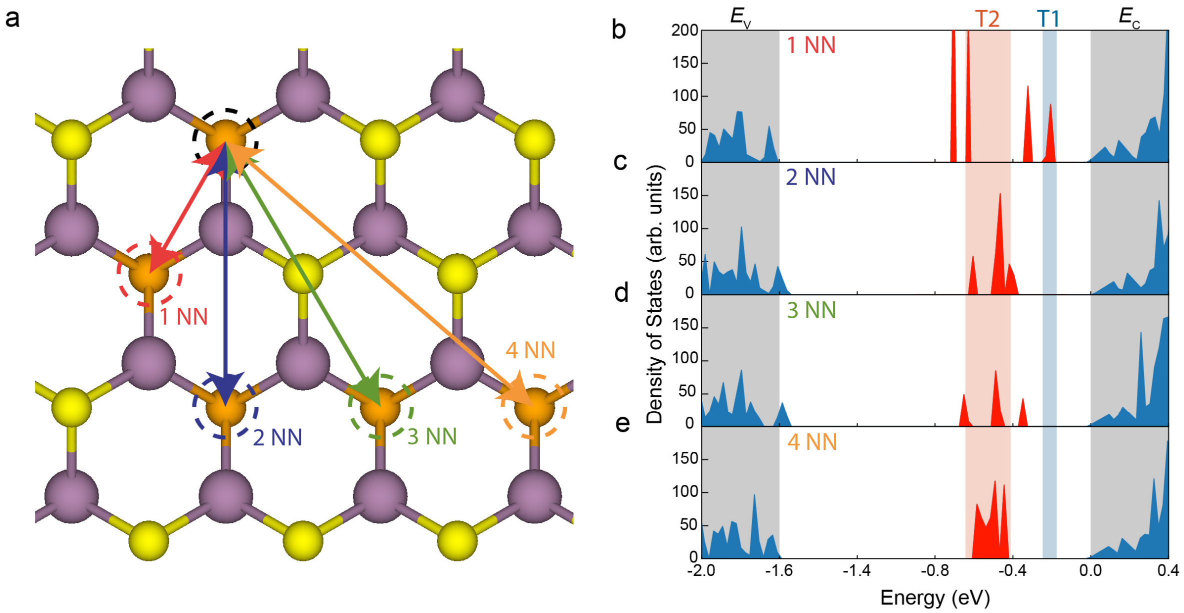

Supplementary Figure 14. Defect hybridization between nearby sulfur vacancies. a) Atomic structures of neighboring sulfur vacancy pairs with different distances. Here 1NN (V_SV_S), 2NN, 3NN, 4NN, respectively, represent two nearby V_S with the closest, the 2nd closest, the 3rd closest, and the 4th closest neighboring atoms. b-e) Density of states (DOS) of the V_S pair defects shown in a). The shaded gray regions mark out the valance and conduction bands. The in-gap defect states are indicated in red and the blue and pink shaded regions are respectively the T1 and T2 defect states measured by DLTS. With increased distance between the V_SV_S pairs, the strong defect hybridization (panel b) starts to mitigate, leading to the final convergence of the four defect levels into two levels resembling the DOS of V_S. Here, the band gap energy E_g is predicted to be 1.68 eV using the PBE functional including spin-orbit coupling.

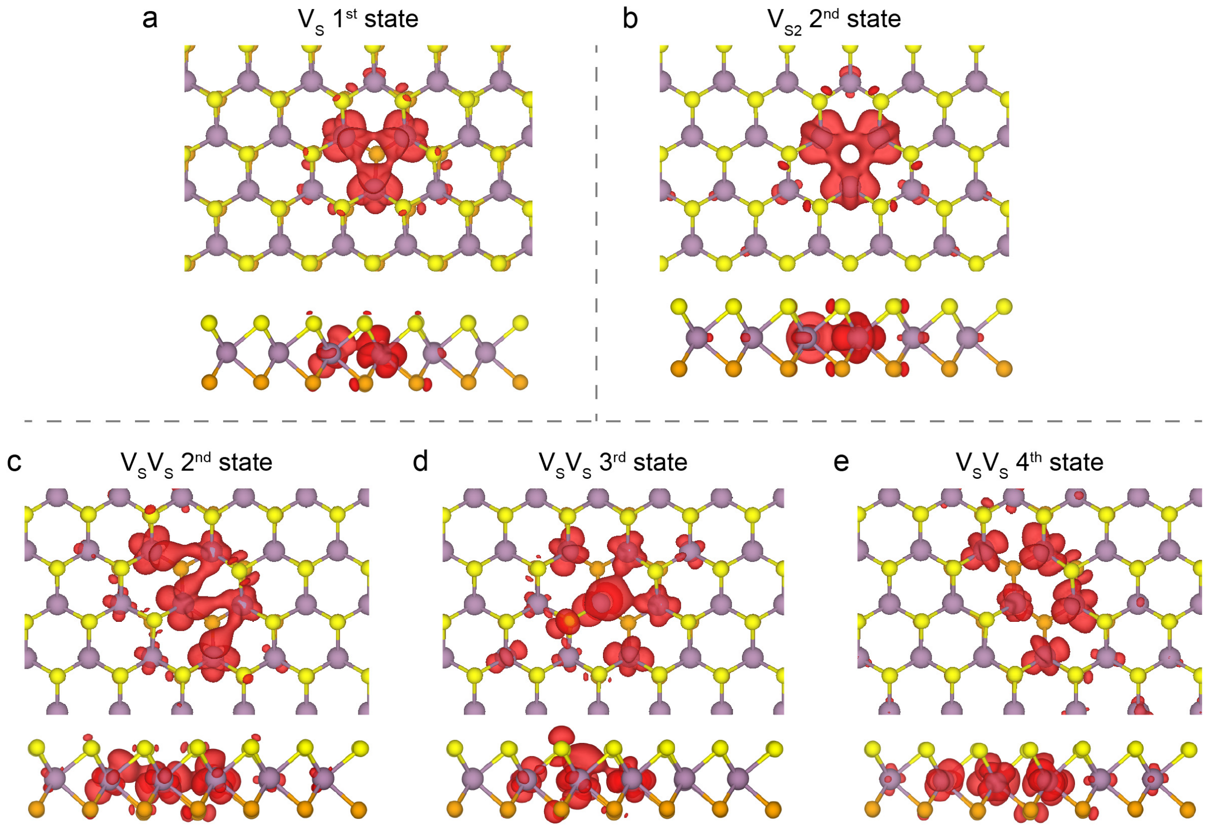

Supplementary Figure 15. Real space charge density of the in-gap defect states. a-b) Top and side view of the charge density distribution of the 1st in-gap defect states for a) V_S and b) V_{S2}. c-e) Top and side view of the charge density distribution of the c) 2nd, d) 3rd and e) 4th in-gap state for neighboring in-plane V_SV_S pairs.

Supplementary Table 1. Trap energy levels of the possible defects in as-grown MoS_2.

Defect type	$E_C - E_{T1}$ (eV)	$E_C - E_{T2}$ (eV)	$E_C - E_{T3}$ (eV)	$E_C - E_T$ for T4 and other deeper levels (eV)
V_S	0.481	0.522		
V_{S2}	0.496	0.578		
V_SV_S (same plane)	0.185	0.306	0.612	0.686
V_SV_S (different plane)	0.109	0.372	0.499	0.838 / 1.537
V_{Mo}	0.719	1.049	1.084	1.416 x 2
S_{Mo}	0.216	0.27	0.881	1.396 / 1.508
$S2_{Mo}$	~0	0.91	1.248	1.316 / 1.366 / 1.62
Mo_S	0.564	0.848	0.91	
Mo_{S2}	0.197	0.231	0.323	0.496 / 1.211 / 1.69
W_{Mo}	-	-	-	-
O_S	-	-	-	-
H_S	0.463	0.498	0.72	0.764
$H2_S$	0.544	-	-	-
$H3_S$ [15]	-	-	-	-
H_{add}	0.115	-	-	-
S_{add}	-	-	-	-

Supplementary Note 11: V_S creation by Ar plasma treatment probed by DLTS

In Supplementary Reference [16], Li *et* al. have conducted a careful study about defect creation in monolayer MoS_2 under mild Ar plasma conditions, where both STEM and XPS measurement confirm the creation of additional sulfur vacancies and the absence of other defect types like Mo vacancies. Here, we first conduct an Ar plasma test to find a mild and non-destructive plasma condition, and then perform the DLTS measurement on a MoS_2 MIS device before and after the mild plasma irradiation (see Methods for details).

Supplementary Figure 16 presents the C-V and DLTS measurement results before and after the plasma irradiation. The high frequency capacitance voltage (HF-CV) curve (Supplementary Figure 16a) retains the same shape as the pristine one but shows a smaller transition slope, a decreased saturation capacitance and an enlarged hysteresis, indicating an increased defect

concentration with preserved electrical properties of MoS_2. According to the isothermal DLTS measurement (Supplementary Figure 16b), an apparent signal enhancement is observed at higher temperatures (300 K and 400 K), further confirming an increased defect concentration after the plasma irradiation. However, detailed defect characteristics, including the trap energy level E_{T} and capture cross section σ_{n}, can only be obtained through a temperature sweep measurement, as shown in Supplementary Figure 16c.

As explained in the Section "Trap state level determination by DLTS" in the main text, the activity of a trap state changes with temperature, where the peaks indicate a matching between the pre-set time window and the trap's time constant at the peak temperature. Here, we observe an apparent increase in the T2 peak intensity, as well as a shift towards higher temperatures. Using the modified Arrhenius function for 2D materials (Eq. 1), we obtain the energy positions of T1 and T2 before and after Ar plasma treatment, as shown in Supplementary Figure 16d. Here, the relative trap concentration, represented by the number of balls, are obtained from the heights of the two peaks in Supplementary Figure 16c. We notice that the shallower trap T1 remains unchanged with respect to both the energy and the trap concentration, while the deeper trap T2 shows an increased defect density of around 25% and a shift of E_{T} towards the mid gap. As illustrated in the section "DFT modeling and hybridized defect states from neighboring vacancies" and Supplementary Figures 14-15, nearby sulfur vacancy pairs (V_SV_S: 1NN, 2NN, 3NN, 4NN) can create additional hybridized defect states in the band gap, where the shortest vacancy distance leads to the largest energy splitting. Accordingly, the downshift of E_{T2} can be explained by a shortened V_SV_S distance in average and thus a stronger hybridization and energy splitting, resulting from the 25% increase of V_S density by the Ar plasma treatment.

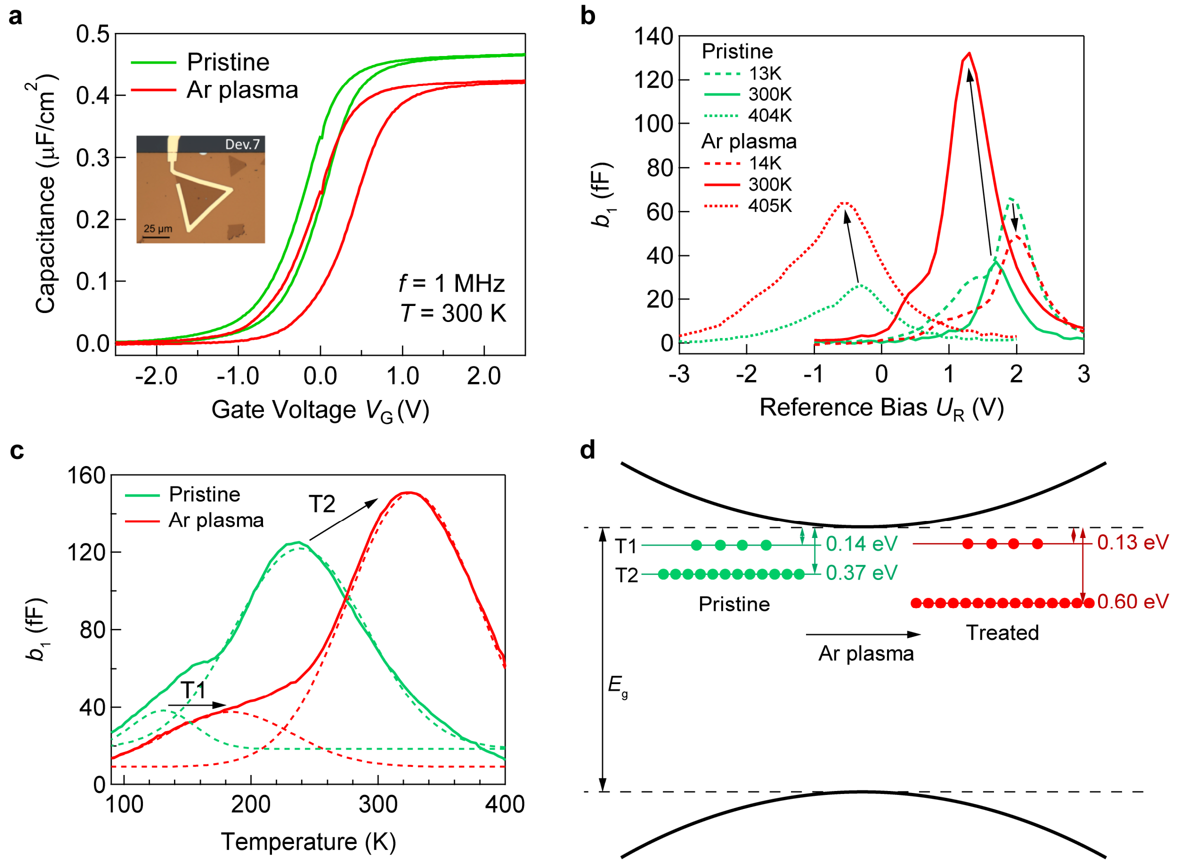

Supplementary Figure 16. C-V and DLTS measurement before and after Ar plasma treatment. a) C-V measurement performed at 300 K and a frequency of 1 MHz. b) Isothermal DLTS measurement with varied reference bias voltage, measured at 14 K, 300 K and 404 K. The pulse height $\boldsymbol{U_H}$, the pulse width $\boldsymbol{t_P}$, and the period width $\boldsymbol{T_W}$ are respectively 3 V, 100 μs, and 100 ms. c) DLTS temperature sweep measurement showing shifted peak position and increased peak amplitude. The reference voltage $\boldsymbol{U_R}$, pulse voltage $\boldsymbol{U_P}$, the pulse width $\boldsymbol{t_P}$, and the period width $\boldsymbol{T_W}$ are respectively 0.5 V, 4.5 V, 100 μs, and 100 ms. d) A schematic diagram showing the relative energy positions and trap concentrations of the two detected defect states in the band gap. The energy position $\boldsymbol{E_T}$ is obtained by performing Arrhenius calculations and linear fittings (Section "DLTS method for monolayer semiconductors"), and the relative trap concentrations are proportional to the peak heights in panel c.

Supplementary Note 12: DLTS measurement of MoS_2 MIS device with a transferred metal contact

According to Supplementary References [17–19], the top contacts patterned using an e-beam evaporator can induce Fermi-level pinning and increased defect concentration. In this paper, the impact of evaporation is significantly reduced by adopting the perimeter contact configuration, where the area of the contact region is ten times smaller than the exposed MoS_2 surface. Furthermore, the e-beam evaporator that we regularly use is characterized by a ~1 m distance between the metal source and the target devices. However, to further testify the

reliability and accuracy of our DLTS methodology, we perform additional experiments using the transferred Au contacts.

Supplementary Figure 17a shows the process flow and optical microscope images during different fabrication steps involving transfer of the metal contact on top of monolayer MoS_2, using the PPC method adapted from Supplementary Reference [18] (see Methods for details). This method is tested several times during the experiment to achieve a complete and well-aligned transfer. To stay consistent, the MoS_2 crystals shown here are from the same MOCVD batch as the previous sections.

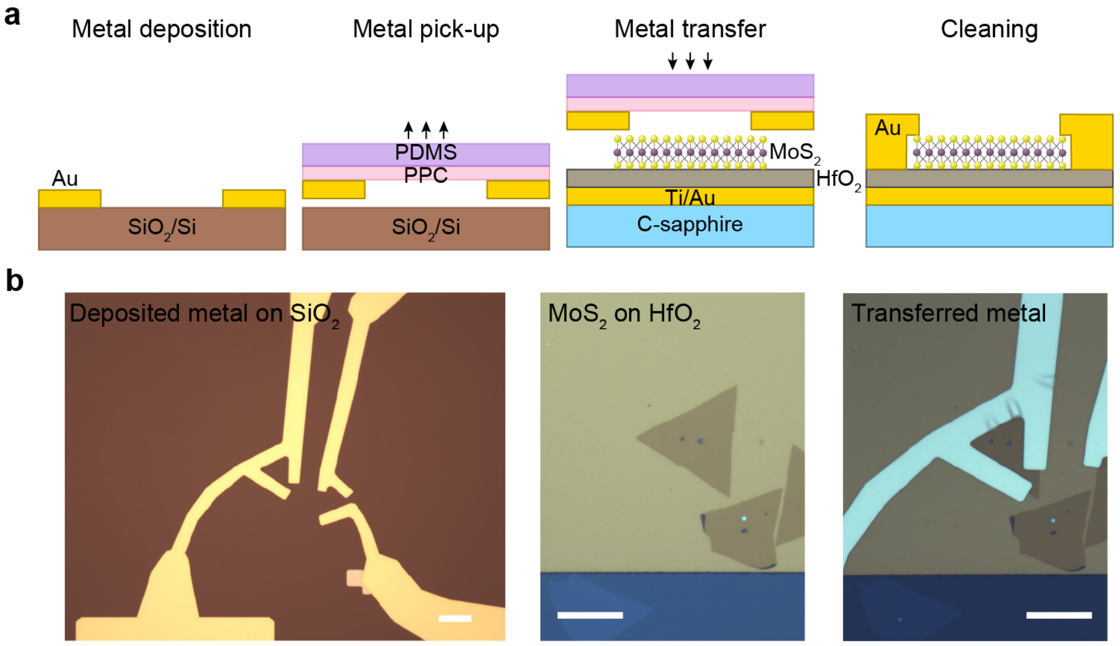

Supplementary Figure 17. Process flow of the fabrication of monolayer MoS_2 MIS device with transferred metal contacts. a) Schematic showing the fabrication process of the PPC/PDMS method of metal transfer. b) Optical microscopy images of the device during the metal transfer process. The scale bar is 25 μm.

After the transfer, the device is loaded into the cryogenic chamber for C-V and DLTS measurements. As shown in Supplementary Figure 18a, the C-V curves with transferred metal contacts exhibit higher positive threshold voltages (V_{TH} from 3.5 V to 5.5 V) than the evaporated contacts (V_{TH} from 0 V to 2.5 V, Supplementary Figure 2). This indicates a reduced Fermi level pinning near the conduction band edge due to the lack of Au–S bonding for the transferred contacts (Supplementary References 17,19). These devices still demonstrate the n-type behavior, but require a higher gate voltage to approach the conduction band.

Supplementary Figure 18b shows the DLTS temperature sweep measurement of a MoS_2 MIS device with transferred contacts, where we again observe our previously demonstrated two peaks at around 100 K and 300 K. Note that the latter is more pronounced and that the former appears as a small tail (marked by a blue arrow). Using different rate windows defined by various correlation functions (Fig. 2a and Supplementary Figure 7), the defect level is determined to be 0.670 eV below the conduction band, corresponding to the T2 level induced by isolated single (V_S) and double (V_{S2}) sulfur vacancies. The low-temperature tail should have a lower activation energy according to the Arrhenius functions (Supplementary Note 5), which is very likely to be associated with neighboring V_SV_S pairs (T1). However, due to the instrumen limitations and the low signal-to-noise ratio, it is difficult to perform an accurate peak fitting and calculate the precise location. These results are consistently observed in another set of devices.

This additional experiment serves as a confirmation that the DLTS measures intrinsic deep-level signals. Together with the defect creation experiment by Ar plasma treatment, all three experiments demonstrate the same dominant DLTS peak associated with the deeper T2 (V_S and V_{S2}) state in our monolayer MoS_2. Meanwhile, the aberration-corrected STEM imaging and theoretical calculations (main text Figs. 4 and 5) also support these results, further confirming the reliability and accuracy of our DLTS methodology.

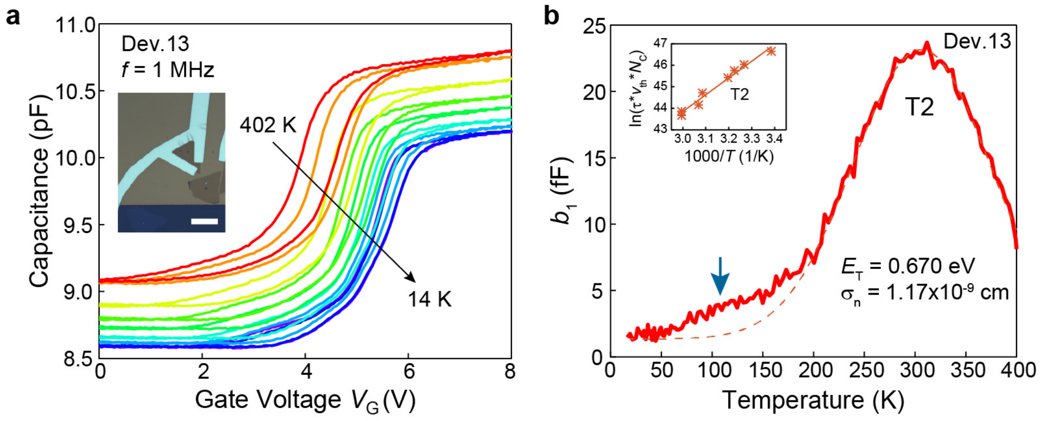

Supplementary Figure 18. C-V and DLTS measurement for a monolayer MoS_2 MIS device with transferred metal contacts. a) Temperature-dependent C-V measurement performed at a frequency of 1 MHz. The inset shows the optical microscope image of the device, with a scale bar of 25 μm. b) DLTS temperature sweep measurement showing a broad DLTS peak corresponding to the T2 trap state.. The reference voltage $\boldsymbol{U_R}$, the pulse voltage $\boldsymbol{U_P}$, the pulse width $\boldsymbol{t_P}$, and the period width $\boldsymbol{T_W}$ are respectively 4.9 V, 8 V, 100 μs, and 1 s. The inset shows the Arrhenius fit obtained from the Fourier transform DLTS signals (Section "DLTS method for monolayer semiconductors").

Supplementary Note 13: DLTS measurement on a control device with MIM configuration

The total capacitance in C-V measurement can be decomposed into three main parts: the parallel stray capacitance C_P between the top electrode and the gate, the quantum capacitance C_Q of monolayer MoS_2, and the geometric oxide capacitance C_g (sometimes also referred to as C_{OX} in the literature). According to Supplementary References [2,20], the parallel stray capacitance C_P originates from the extra area of the top electrode not covering the MoS_2 crystals, and therefore can be directly subtracted as a background from the C-V curve. In addition, the oxide capacitance (C_g or C_{OX}) is a constant value and can be directly calculated using the flake area, the oxide permittivity and thickness. Therefore, only MoS_2 capacitance is tuned by the gate voltage, whose transient signal contributes to the DLTS spectra. However, to further confirm the origin of the DLTS signal and exclude any influence from the oxide, top electrode or bottom gate, we design a control experiment using the metal-insulator-metal (MIM) structure without the monolayer MoS_2 channel. We also use the same surrounding-contact configuration to stay consistent.

Supplementary Figure 19 shows the C-V and DLTS measurements performed on the control MIM device. In Supplementary Figure 19a, the device capacitance remains constant for all frequencies and applied gate voltages, with slight changes in the noise level. In Supplementary Figure 19b we show the isothermal DLTS measurement with fixed pulse height U_{H} and varied reference voltage U_{R}. This way, any transient response at different gate voltages can be detected. As expected, we did not observe any significant DLTS signals for both high and low temperatures. Similarly, no visible DLTS peak is observed in the following DLTS temperature sweep measurement (Supplementary Figure 19c). The exact measurements are also performed on two other devices, and the results are very similar. These results confirm that the oxide and parasitic capacitances have constant values in our measurements, within the applied frequency from 2 kHz to 1 MHz, the gate voltage from -8 V to 8 V and the temperature from 14 K to 410 K. Therefore, they do not contribute to the DLTS signals and peaks shown in the previous sections, and we conclude that the DLTS spectra solely originate from the MoS_2 capacitance.

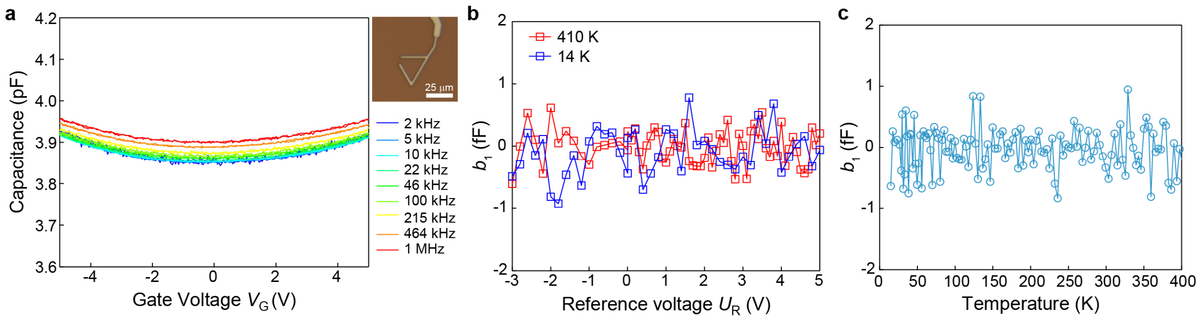

Supplementary Figure 19. C-V and DLTS measurement for a control MIM device without MoS_2 layer. a) Frequency-dependent C-V curves measured at room temperature. The optical microscope image of the control device is shown on the right side. b) DLTS isothermal measurement performed at 410 K and 14 K, showing no DLTS signal from the device geometry or the oxide. The pulse height $\boldsymbol{U}_{\mathbf{H}}$, the pulse width $\boldsymbol{t}_{\mathbf{P}}$, and the period width $\boldsymbol{T}_{\mathbf{W}}$ are respectively 3 V, 100 µs, and 1 s. c) DLTS temperature sweep measurement for the control device, showing only background noise without visible DLTS peaks. The reference voltage $\boldsymbol{U}_{\mathbf{R}}$, pulse voltage $\boldsymbol{U}_{\mathbf{P}}$, the pulse width $\boldsymbol{t}_{\mathbf{P}}$, and the period width $\boldsymbol{T}_{\mathbf{W}}$ are respectively 1 V, 4 V, 100 µs, and 1 s.

Supplementary References

1. Ma, N. & Jena, D. Carrier statistics and quantum capacitance effects on mobility extraction in two-dimensional crystal semiconductor field-effect transistors. *2D Mater.* **2**, 015003 (2015).
2. Fang, N. & Nagashio, K. Band tail interface states and quantum capacitance in a monolayer molybdenum disulfide field-effect-transistor. *J. Phys. Appl. Phys.* **51**, 065110 (2018).
3. Gaur, A. *et al.* Analysis of admittance measurements of MOS capacitors on CVD grown bilayer MoS_2. *2D Mater.* **6**, 035035 (2019).
4. Gaur, A. *et al.* A MOS capacitor model for ultra-thin 2D semiconductors: the impact of interface defects and channel resistance. *2D Mater.* **7**, 035018 (2020).
5. Zhu, W. *et al.* Electronic transport and device prospects of monolayer molybdenum disulphide grown by chemical vapour deposition. *Nat. Commun.* **5**, 3087 (2014).
6. Lang, D. V. Deep-level transient spectroscopy: A new method to characterize traps in semiconductors. *J. Appl. Phys.* **45**, 3023–3032 (1974).
7. Yun, W. S., Han, S. W., Hong, S. C., Kim, I. G. & Lee, J. D. Thickness and strain effects on electronic structures of transition metal dichalcogenides: 2H-MX_2 semiconductors (M = Mo, W; X = S, Se, Te). *Phys. Rev. B* **85**, 033305 (2012).
8. Huang, S. *et al.* Capture and emission mechanisms of defect states at interface between nitride semiconductor and gate oxides in GaN-based metal-oxide-semiconductor power transistors. *J. Appl. Phys.* **126**, 164505 (2019).
9. Hafner, J. Ab-initio simulations of materials using VASP: Density-functional theory and beyond. *J. Comput. Chem.* **29**, 2044–2078 (2008).
10. Zhou, W. *et al.* Intrinsic Structural Defects in Monolayer Molybdenum Disulfide. *Nano Lett.* **13**, 2615–2622 (2013).
11. Qiu, H. *et al.* Hopping transport through defect-induced localized states in molybdenum disulphide. *Nat. Commun.* **4**, 2642 (2013).
12. KC, S., Longo, R. C., Addou, R., Wallace, R. M. & Cho, K. Impact of intrinsic atomic defects on the electronic structure of MoS_2monolayers. *Nanotechnology* **25**, 375703 (2014).
13. Zhu, Z. Y., Cheng, Y. C. & Schwingenschlögl, U. Giant spin-orbit-induced spin splitting in two-dimensional transition-metal dichalcogenide semiconductors. *Phys. Rev. B* **84**, 153402 (2011).
14. Barja, S. *et al.* Identifying substitutional oxygen as a prolific point defect in monolayer transition metal dichalcogenides. *Nat. Commun.* **10**, 3382 (2019).
15. Lu, H., Kummel, A. & Robertson, J. Passivating the sulfur vacancy in monolayer MoS_2. *APL Mater.* **6**, 066104 (2018).
16. Li, H. *et al.* Activating and optimizing MoS_2 basal planes for hydrogen evolution through the formation of strained sulphur vacancies. *Nat. Mater.* **15**, 48–53 (2016).
17. Liu, Y. *et al.* Approaching the Schottky–Mott limit in van der Waals metal–semiconductor junctions. *Nature* **557**, 696–700 (2018).
18. Jung, Y. *et al.* Transferred via contacts as a platform for ideal two-dimensional transistors. *Nat. Electron.* **2**, 187–194 (2019).
19. Popov, I., Seifert, G. & Tománek, D. Designing Electrical Contacts to MoS_2 Monolayers: A Computational Study. *Phys. Rev. Lett.* **108**, 156802 (2012).
20. Chen, X. *et al.* Probing the electron states and metal-insulator transition mechanisms in molybdenum disulphide vertical heterostructures. *Nat. Commun.* **6**, 6088 (2015).
